# Mycophenolate Mofetil versus Cyclophosphamide for Initial Therapy in Childhood-Onset Proliferative Lupus Nephritis: A Prospective, Multicenter, Randomized Trial

**DOI:** 10.1681/ASN.0000000866

**Published:** 2025-09-12

**Authors:** Ying Wang, Xiaoyan Li, Shan Jian, Jing Li, Junxia Yan, Shuzhen Sun, Zhenle Yang, Weimin Zheng, Qun Li, Qi Zheng, Meiping Lu, Mo Wang, Qin Yang, Huawei Mao, Tongxin Han, Yi Lin, Qiuye Zhang, Yue Du, Ying Tang, Yong Cai, Liangzhong Sun, Jianjiang Zhang, Junmei Liu, Zanhua Rong, Lijun Jiang, Haitao Bai, Yan Chen, Jun Yang, Linlin Wang, Wei Zhang, Xinyi Wei, Yun Zhu, Xiaozhong Li, Xingyuan Xie, Dujuan Zhou, Yongzhen Li, Yan Cao, Tian Shen, Qian Liu, Hongmei Song, Xiaochuan Wu

**Affiliations:** 1 Department of Pediatrics, The Second Xiangya Hospital, Central South University, Changsha, China; 2 Department of Pediatrics, Peking Union Medical College Hospital, Chinese Academy of Medical Sciences and Peking Union Medical College, Beijing, China; 3 Department of Rheumatology and Clinical Immunology, Peking Union Medical College Hospital, Chinese Academy of Medical Sciences and Peking Union Medical College, Beijing, China; 4 Department of Epidemiology and Health Statistics and Hunan Provincial Key Laboratory of Clinical Epidemiology, Xiangya School of Public Health, Central South University, Changsha, China; 5 Department of Pediatric Nephrology and Rheumatology and Immunology, Shandong Provincial Hospital Affiliated to Shandong First Medical University, Jinan, China; 6 Department of Nephrology, Jiangxi Provincial Children's Hospital, Nanchang, China; 7 Department of Rheumatology Immunology and Allergy, Children's Hospital, Zhejiang University School of Medicine, Hangzhou, China; 8 Department of Nephrology, Children's Hospital of Chongqing Medical University, Chongqing, China; 9 Department of Rheumatology and lmmunology, Beijing Children's Hospital, National Center for Children's Health, Capital Medical University, Beijing, China; 10 Department of Pediatric Nephrology and Rheumatology, Affiliated Hospital of Qingdao University, Qingdao, China; 11 Department of Pediatrics, Shengjing Hospital of China Medical University, Shenyang, China; 12 Department of Pediatrics, Nanfang Hospital, Southern Medical University, Guangzhou, China; 13 Department of Pediatrics, the First Affiliated Hospital of Zhengzhou University, Zhengzhou, China; 14 Department of Pediatrics, the Second Hospital of Hebei Medical University, Shijiazhuang, China; 15 The First Affiliated Hospital of Xiamen University, Xiamen, China; 16 Department of Rheumatology and Immunology, Shenzhen Children's Hospital, Shenzhen, China; 17 Pediatric Immunology and Rheumatology Department, Chengdu Women's and Children's Central Hospital, School of Medicine, University of Electronic Science and Technology of China, Chengdu, China; 18 Department of Nephrology and Immunology, Children's Hospital of Soochow University, Suzhou, China; 19 Department of Pediatric Nephrology, Rheumatology and Immunology, the First People's Hospital of Chenzhou, Chenzhou, China

**Keywords:** chronic GN, chronic nephropathy, chronic kidney disease, clinical nephrology, clinical trial, immunosuppression, lupus nephritis, pediatric nephrology, pediatrics, SLE

## Abstract

**Key Points:**

- The total renal response rate in the mycophenolate mofetil group was found to be noninferior to that in the cyclophosphamide group.
- There was no significant difference in the incidence of adverse drug reactions between the mycophenolate mofetil and cyclophosphamide groups.
- The reduction in SLE Disease Activity Index scores was similar between the two groups.

**Background:**

Recent studies suggest that oral mycophenolate mofetil (MMF) may be similar to intravenous cyclophosphamide in treating lupus nephritis. However, these therapies have not been prospectively compared in childhood-onset lupus nephritis.

**Methods:**

In this prospective, multicenter, randomized trial, patients aged 5–17 years with proliferative lupus nephritis (class 3/4±5) and severely increased proteinuria (urine protein-creatinine ratio ≥1000 mg/g and/or 24-hour urinary protein excretion >25 mg/kg) were randomly assigned to receive either MMF or intravenous cyclophosphamide as initial therapy, alongside glucocorticoids. The primary end point was total renal response (TRR) at 24 weeks, with the aim of demonstrating the noninferiority of MMF compared with intravenous cyclophosphamide, using a noninferiority margin of 12%. TRR encompassed complete renal response, primary efficacy renal response, and partial renal response. Secondary end points assessed systemic disease activity and safety.

**Results:**

A total of 107 patients were enrolled from 17 hospitals, with 52 assigned to the MMF group (47 completed the 24-week therapy) and 55 assigned to the cyclophosphamide group (48 completed the 24-week therapy). In the intention-to-treat population, the TRR rate was 92% in the MMF group and 89% in the cyclophosphamide group (test for noninferiority, *P* = 0.008). In the per-protocol population, renal response was observed in 96% of patients in the MMF group versus 94% of patients in the cyclophosphamide group (test for noninferiority, *P* = 0.009). The difference in TRR rate between the MMF and cyclophosphamide groups was 3% (95% confidence interval, −9% to 15%) in the intention-to-treat population and 2% (95% confidence interval, −9% to 13%) in the per-protocol population. There were no significant differences in the incidence of adverse drug reactions between the MMF and cyclophosphamide groups in the intention-to-treat population (10% versus 15%, continuity correction chi-squared test, *P* = 0.44).

**Conclusions:**

After 24 weeks of therapy, oral MMF was noninferior to intravenous cyclophosphamide as initial therapy for childhood-onset proliferative lupus nephritis and exhibited a similar safety profile.

**Clinical Trial registry name and registration number:**

MMF versus cyclophosphamide in the Induction Therapy of Pediatric Active Proliferative lupus nephritis, ClinicalTrials.gov, NCT05495893.

## Visual Abstract

**Figure:**
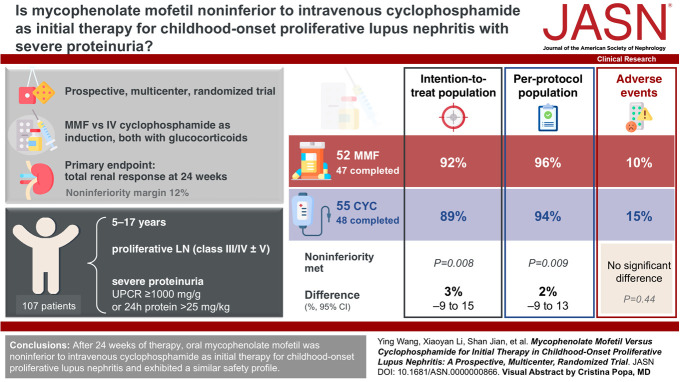

## Introduction

SLE is a chronic, systemic, multiorgan autoimmune disease with diverse clinical manifestations.^1^ SLE is particularly prevalent and severe among Asian, African, and Hispanic populations.^2^ Lupus nephritis, one of the most severe organ manifestations, occurs in 12%–69% of patients with SLE in different populations.^2,3^ Lupus nephritis is a negative prognostic factor that fundamentally influences the prognosis of SLE. Despite decades of treatment advancements, lupus nephritis remains the leading cause of mortality in SLE, with 5%–20% of lupus nephritis patients developing kidney failure after 10 years.^4,5^ Proliferative lupus nephritis, classified under the International Society of Nephrology (ISN)/Renal Pathology Society (RPS)^6^ classes 3, 4, 3+5, and 4+5, is usually symptomatic, more aggressive, and can lead to AKI, resulting in permanent nephron loss and premature death if not treated effectively.^7,8^

Dual immunosuppressive regimens comprising glucocorticoids and cyclophosphamide have been the standard-of-care initial therapy for active proliferative lupus nephritis for decades.^7^ However, the risks of infertility and future hematologic malignancies associated with cyclophosphamide have prompted exploration of alternative initial regimens. Since its approval for the prevention of transplant rejection, many studies have focused on mycophenolate mofetil (MMF) as initial therapy for lupus nephritis. Several previous studies on lupus nephritis have shown that MMF is as effective as intravenous cyclophosphamide in treating active lupus nephritis.^8–11^ Although the use of MMF does not predispose patients to gonadal failure or hematologic malignancies, the rates of other adverse events related to MMF and intravenous cyclophosphamide have been inconsistently reported. In the Aspreva Lupus Management Study, no significant differences were observed between the MMF and intravenous cyclophosphamide groups for adverse events, serious adverse events, or infections.^9^ However, some studies have shown that gastrointestinal symptoms occur significantly more frequently in patients receiving MMF than in those receiving cyclophosphamide.^10,12^

Childhood-onset SLE not only presents a higher proportion of cases developing lupus nephritis compared with adult-onset SLE but also tends to manifest more severe disease forms in pediatric populations.^13–16^ In 2017, the Single Hub and Access Point for Pediatric Rheumatology in Europe initiative^17^ recommended MMF or intravenous cyclophosphamide combined with glucocorticoids as the initial regimen for proliferative lupus nephritis in pediatric patients, a recommendation largely based on clinical trials in adults and few retrospective observational studies in children.^18^ To date, no adequately powered randomized controlled trials (RCTs) have directly compared initial regimens for childhood-onset lupus nephritis. Current evidence remains insufficient to confirm the superior efficacy of either treatment regimen, highlighting a significant unmet need for identifying optimal therapies in this population.

In this open-label, multicenter, randomized, noninferiority study, we compared the efficacy and safety of oral MMF plus glucocorticoids versus intravenous cyclophosphamide plus glucocorticoids as initial therapy for renal remission in newly diagnosed pediatric patients with proliferative lupus nephritis who had an eGFR ≥60 ml/min per 1.73 m^2^ and either a urine protein-creatinine ratio (UPCR) ≥1000 mg/g or 24-hour urinary protein excretion >25 mg/kg.

## Methods

### Trial Design

This multicenter, prospective, randomized, open-label interventional study was conducted at 17 Chinese medical institutions from June 2022 to November 2024. Using a two-arm, parallel-group design with 1:1 participant allocation, the study enrolled participants who were followed for 24 weeks. The protocol received ethical approval from the Medical Ethics Committee of the Second Xiangya Hospital of Central South University (Approval No. 2022-063), and written informed consent was obtained from all participants before enrollment.

### Participants

Eligible patients were newly diagnosed with SLE and met all the following inclusion criteria: (*1*) age 5–17 years at enrollment; (*2*) diagnosed with SLE according to the 2019 American College of Rheumatology classification criteria^19^ or the 2012 Systemic Lupus International Collaborating Clinics criteria^20^; (*3*) biopsy-proven proliferative lupus nephritis (ISN/RPS 2003 class 3/4±5); (*4*) eGFR ≥60 ml/min per 1.73 m^2^ with severely increased proteinuria (spot urine UPCR ≥1000 mg/g and/or 24-hour urinary protein excretion ≥25 mg/kg), blood white blood cell count ≥3.0×10^9^/L, and lymphocyte count ≥0.5×10^9^/L; and (*5*) no prior use of immunosuppressants or biologics before inclusion. The exclusion criteria included a history of diseases predisposing to infections, active or historical malignancy, severe infections, serious SLE complications (*e.g*., lupus encephalopathy, diffuse alveolar hemorrhage, hemoglobin <60 g/L, platelet count <10.0×10^9^/L), unstable vital signs, and allergies to cyclophosphamide, MMF, or glucocorticoids (see Supplemental Material for full eligibility criteria).

### Randomization and Interventions

Eligible patients were randomly assigned to either the MMF or intravenous cyclophosphamide groups. Randomization was performed using a computer-generated random number list with a 1:1 allocation ratio, and the allocation was concealed in numbered, sealed, and opaque envelopes.

All patients in the cyclophosphamide group received monthly intravenous cyclophosphamide therapy (750 mg/m^2^, not exceeding 1 g, administered over 1–2 days) for 6 months combined with glucocorticoids. All patients in the MMF group received MMF therapy (30–40 mg/kg per day, divided into two doses, not exceeding 1 g/dose) in conjunction with glucocorticoids. The initial therapy phase was defined as 24 weeks, as the response at 24 weeks is associated with disease outcome.^9^ The glucocorticoids treatment was standardized in accordance with the “International Consensus for The Dosing of Corticosteroids in Childhood-Onset SLE with Proliferative Lupus Nephritis.”^21^ All patients received intravenous pulses of methylprednisolone (15–30 mg/kg per day, not exceeding 500 mg/dose, administered 3 days per week for 2 weeks), followed by oral prednisone at 2 mg/kg per day (not exceeding 60 mg/d), which was then rapidly tapered by 24 weeks. All patients were given supportive therapy including the hydroxychloroquine (4–6 mg/kg per day) and angiotensin-converting enzyme inhibitors.

### Data Collection and End Points

A standardized data collection form was used for all eligible patients at baseline, including information such as sex, age at enrollment, disease duration, kidney pathology, serum creatinine (eGFR according to the Schwartz formula), double-stranded DNA antibody, complement C3, complement C4, urinalysis (hematuria was defined as >5 red blood cell/high-power field by manual microscopy),^22^ SLE Disease Activity Index 2000 (SLEDAI-2K) score, and evaluation of adverse events. Proteinuria and serum creatinine were serially assessed at weeks 4, 8, 12, and 24.

The primary end point was the proportion of the intention-to-treat populations who achieved a renal response to treatment at 24-week milestones, defined as the composite of complete renal response (CRR), primary efficacy renal response, and partial renal response (PRR). The definitions of treatment response in lupus nephritis were based on the Kidney Disease Improving Global Outcomes 2024 Clinical Practice Guideline for the Management of Lupus Nephritis.^7^ CRR was defined as proteinuria <0.5 g/1.73 m^2^ per day or  <300 mg/m^2^ per day based on a 24-hour urine specimen, along with stabilization or improvement in kidney function (±10%–15% of baseline). Primary efficacy renal response was defined as a urine UPCR <700 mg/g and eGFR no worse than 20% below the preflare value or  ≥60 ml/min per 1.73 m^2^. PRR was defined as a reduction in proteinuria by at least 50%, UPCR <3000 mg/g, and stabilization or improvement in kidney function (±10%–15% of baseline). No renal response was defined as failure to achieve either a partial or CRR. Key secondary end points included the change in the SLEDAI-2K score from baseline and the assessment of safety (including infections, menstrual disorders, and hematologic abnormalities, among other adverse events). Exploratory end points comprised renal response at week 12 and *post hoc* subgroup noninferiority analyses of renal response at week 24, including: patients with nephrotic-range proteinuria, those with ISN/RPS class 4 or 4±5 histology, case with proliferative and membranous features (class 3±5/4±5), and those with pure proliferative lupus nephritis (class 3/4).

### Sample Size Calculation

**Figure 1:**
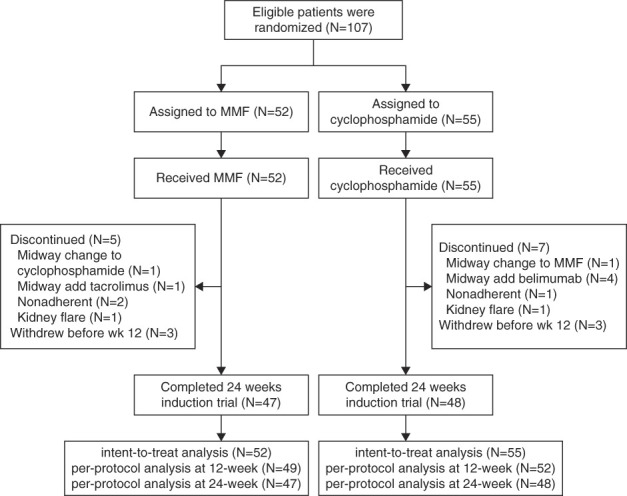
**Patient flow and disposition.** MMF, mycophenolate mofetil.

Based on a published meta-analysis focusing on the treatment outcomes of Chinese patients with lupus nephritis,^23^ we considered the total renal response (TRR) rate (complete+primary efficacy+partial responses) to be 84% in the MMF group and 71% in the cyclophosphamide group. The sample size was calculated to demonstrate noninferiority of MMF versus intravenous cyclophosphamide for TRR rates at 24 weeks, using the following parameters: one-sided type 1 error of 0.025, 80% power, and a noninferiority margin of 12%. This calculation yielded a required sample size of 88 patients (44 per group). Accounting for a 15% patient withdrawal rate, the adjusted total sample size was calculated as 104. Ultimately, 107 patients were enrolled, with 95 patients completing the study (Figure 1).

### Statistical Methods

Patient characteristics were summarized using descriptive statistics: continuous variables as mean±SD or median (25–75th percentile) and categorical variables as counts (proportions). Between-group comparisons were reported as mean differences (MDs) or rate differences (RDs) with corresponding 95% confidence intervals (CIs), calculated using *t* tests and the Newcombe–Wilson method, respectively. The noninferiority of TRR and CRR rates between the two groups was assessed using Farrington–Manning testing, with a 12% noninferiority margin in both intention-to-treat and per-protocol populations. Missing data were handled through last observation carried forward for intention-to-treat analysis. Adverse drug reactions were analyzed using chi-squared tests for categorical variables. Statistical analysis was performed using Statistical Analysis System software (version 9.4; SAS Institute, Cary, NC), and SPSS Statistics v.26.0 (IBM Corp., Armonk, NY) was used.

## Results

### Patient Disposition and Characteristics

The study enrolled 107 pediatric patients from 17 centers, with 52 participants randomized to the MMF group and 55 participants randomized to the cyclophosphamide group. The median age of all intention-to-treat population was 12 years (interquartile range, 10–13 years; range, 5–16 years). The demographics and baseline characteristics of intention-to-treat population are presented in Table 1. Overall treatment adherence was excellent, with 89% (95/107) of participants completing the 24-week follow-up. Withdrawal rates were similarly low in both treatment groups, with 10% (five patients) in the MMF group and 13% (seven patients) in the cyclophosphamide group. The baseline characteristics of per-protocol analyzable patients are presented in Supplemental Table 1. Reasons for withdrawal before 24 weeks are illustrated in Figure 1.

**Table 1:** Baseline characteristics of the intention-to-treat population in a trial of pediatric proliferative lupus nephritis

Characteristics	MMF Group (*N*=52)	Cyclophosphamide Group (*N*=55)
Age at enrollment, yr, median (Q1–Q3)	12 (9–13)	12 (10–13)
Male, no. (%)	12 (23)	12 (22)
Duration of disease, d, median (Q1–Q3)	29 (12–60)	25 (10–50)
eGFR, ml/min per 1.73 m^2^, median (Q1–Q3)	102 (72–132)	92 (71–128)
BUN level, mg/dl, median (Q1–Q3)	18 (13–26)	22 (16–31)
Serum albumin level, g/dl, mean (SD)	2.7 (0.7)	2.7 (0.7)
Hemoglobin level, g/dl, mean (SD)	10.3 (2.2)	10.2 (2.0)
24-h urine protein, mg, median (Q1–Q3)	2885 (1475–4360)	3000 (1410–5815)
Nephrotic-range proteinuria, no. (%)	36 (69)	38 (69)
Hematuria, no. (%)	41 (79)	46 (84)
Hypertension, no. (%)	21 (40)	27 (49)
**Kidney biopsy class, no. (%)**		
3 or 4	28 (54)	33 (60)
3+5 or 4+5	24 (46)	22 (40)
Low C3, no. (%)	51 (98)	53 (96)
Low C4, no. (%)	45 (87)	47 (85)
dsDNA antibody positive, no. (%)	49 (94)	50 (91)
SLEDAI-2K score, mean (SD)	18 (5)	18 (5)

Data are presented as *n* (%), median (25–75th percentile), or mean±SD. dsDNA, double-stranded DNA; MMF, mycophenolate mofetil; SLEDAI-2K, SLE Disease Activity Index 2000.

### Primary Outcome: Renal Response

#### Total Renal Response Rate

Among the intention-to-treat population, the 24-week TRR rates were 92% (48 of 52) for the MMF group and 89% (49 of 55) for the cyclophosphamide group, showing a RD of 3% (95% CI, −9% to 15%; noninferiority test, *P* = 0.008) (Table 2). The data for the per-protocol population were consistent with those observed for the intention-to-treat population. The TRR was observed in 96% (45 of 47) of patients in the MMF group and 94% (45 of 48) of patients in the cyclophosphamide group at 24 weeks (noninferiority test, *P* = 0.009), with a TRR RD of 2% (95% CI, −9% to 13%) in the per-protocol population (Table 3).

**Table 2:** Primary and secondary end points at week 24 (intention-to-treat population)

End Point	MMF Group (*N*=52)	Cyclophosphamide Group (*N*=55)	Treatment Difference (95% CI)^a^
Total renal response, no. (%)	48 (92)	49 (89)	3 (−9 to 15)
Complete renal response, no. (%)	40 (77)	38 (69)	8 (−9 to 24)
Primary efficacy renal response, no. (%)	3 (6)	4 (7)	−1 (−12 to 9)
Partial renal response, no. (%)	5 (10)	7 (13)	−3 (−16 to 10)
No renal response, no. (%)	4 (8)	6 (11)	−3 (−15 to 9)
SLEDAI-2K change from baseline, mean (SD)	12 (6)	13 (7)	−1 (−3 to 2)

CI, confidence interval; MMF, mycophenolate mofetil; SLEDAI-2K, SLE Disease Activity Index 2000.

a The treatment difference is given as rate difference or mean difference and corresponding 95% confidence intervals.

**Table 3:** Primary and secondary end points at week 24 (per-protocol population)

End Point	MMF Group (*N*=47)	Cyclophosphamide Group (*N*=48)	Treatment Difference (95% CI)^a^
Total renal response, no. (%)	45 (96)	45 (94)	2 (−9 to 13)
Complete renal response, no. (%)	38 (81)	36 (75)	6 (−11 to 22)
Primary efficacy renal response, no. (%)	3 (6)	2 (4)	2 (−8 to 13)
Partial renal response, no. (%)	4 (9)	7 (15)	−6 (−20 to 8)
No renal response, no. (%)	2 (4)	3 (6)	−2 (−13 to 9)
SLEDAI-2K change from baseline, mean (SD)	13 (5)	14 (6)	−1 (−3 to 2)

CI, confidence interval; MMF, mycophenolate mofetil; SLEDAI-2K, SLE Disease Activity Index 2000.

a The treatment difference is given as rate difference or mean difference and corresponding 95% confidence intervals.

#### Complete Renal Response Rate

The intention-to-treat analysis showed that the CRR rates in the MMF group and cyclophosphamide group were 77% (40 of 52) and 69% (38 of 55), respectively (RDs, 8%; 95% CI, −9% to 24%; noninferiority test, *P* = 0.01) at 24-week (Table 2). In addition, the per-protocol analysis showed that CRR was achieved in 38 of 47 (81%) patients in the MMF group and 36 of 48 (75%) patients in the cyclophosphamide group at 24 weeks. The difference in CRR rate between the two groups was estimated to be 6% (95% CI, −11% to 22%; noninferiority test, *P* = 0.02) in the per-protocol population (Table 3).

### Secondary Outcomes

#### Disease Activity

The SLEDAI-2K score changes from baseline to week 24 showed no significant differences between MMF and cyclophosphamide groups in both intention-to-treat population (MD±SEM = −1 ± 1; 95% CI, −3 to 2; *t* test, *P* = 0.61; Table 2) and per-protocol population (MD±SEM = −1 ± 1, 95% CI, −3 to 2, *t* test, *P* = 0.79; Table 3).

#### Adverse Events

Among patients who withdrew from the MMF group, two did so due to unsatisfactory treatment efficacy: one because of persistent extrarenal manifestations (requiring tacrolimus add-on therapy) and the other due to recurrent proteinuria. Similarly, in the cyclophosphamide group, two patients withdrew because of unsatisfactory efficacy: one owing to failure to achieve PRR by week 12 and the other because of recurrent proteinuria. These events were classified as serious adverse events, with comparable incidence rates (4% each) between the groups. In addition, mild extrarenal lupus flares occurred in four MMF group patients (manifestations: rash [*n*=3], hematologic abnormalities [*n*=2], arthralgia [*n*=1], and serositis [*n*=1]) and three cyclophosphamide group patients (rash [*n*=1], fever [*n*=1], arthralgia [*n*=1], and serositis [*n*=1]).

A total of 13 patients experienced adverse drug reactions, with five of 52 patients (10%) in the MMF group and eight of 55 patients (15%) in the cyclophosphamide group reporting such events (continuity correction chi-squared test, *P* = 0.44). The most common adverse event was infection. The second most common adverse reactions were hematologic. Specifically, in the MMF group, there was one case of decreased CD4^+^ T-cell count. In the cyclophosphamide group, one case of leukopenia and two cases of reduced lymphocyte counts were observed. In addition, one case of menstrual irregularity occurred in the cyclophosphamide group (Table 4). Gastrointestinal complaints were surprisingly rare in our patients.

**Table 4:** Adverse drug reactions in intention-to-treat population

Adverse Drug Reaction	MMF Group (*N*=52)	Cyclophosphamide Group (*N*=55)
Total, no. (%)	5 (10)	8 (15)
Infection, no. (%)	4 (8)	4 (7)
Hematologic adverse, no. (%)	1 (2)	3 (5)
Menstrual irregularity, no. (%)	0 (0)	1 (2)

MMF, mycophenolate mofetil.

### Exploratory Outcomes

#### Treatment Response at 12 Weeks

At week 12, the TRR rate in the MMF group was noninferior to that in the cyclophosphamide group, in both the intention-to-treat population (noninferiority test, *P* = 0.009) and per-protocol population (noninferiority test, *P* = 0.003). Similarly, the CRR rate in MMF group demonstrated noninferiority to cyclophosphamide group in both the intention-to-treat (noninferiority test, *P* = 0.02) and per-protocol (noninferiority test, *P* = 0.01) analyses. No significant differences occurred in SLEDAI-2K score reduction from baseline to week 12 between two groups in the intention-to-treat (*t* test, *P* = 0.56) and per-protocol (*t* test, *P* = 0.91) population (Supplemental Tables 2 and 3).

#### Renal Response of Different Subgroups

The subgroup analysis among participants with nephrotic-range proteinuria revealed that 74 and 65 patients exhibited nephrotic-range proteinuria in the intention-to-treat and per-protocol population, respectively (Supplemental Tables 4 and 5). In both intention-to-treat and per-protocol populations, the MMF group demonstrated noninferiority to the cyclophosphamide group for both TRR rates (intention-to-treat analysis, noninferiority test, *P* = 0.01; per-protocol analysis, noninferiority test, *P* = 0.02) and CRR rates (intention-to-treat analysis, noninferiority test, *P* = 0.01; per-protocol analysis, noninferiority test, *P* = 0.02) at 24 weeks.

Histologic subgroup analysis revealed that most patients (79%) diagnosed with class 4 or 4+5 lupus nephritis. Within this subgroup, the MMF group demonstrated noninferiority to cyclophosphamide group for both TRR (intention-to-treat analysis, noninferiority test, *P* = 0.01; per-protocol analysis, noninferiority test, *P* = 0.02) and CRRs (intention-to-treat analysis, noninferiority test, *P* = 0.01; per-protocol analysis, noninferiority test, *P* = 0.03). A combination of membranous and proliferative (class 3+5 or 4+5) lupus nephritis was identified in 46 patients in the intention-to-treat population and 41 patients in the per-protocol population. Among patients exhibiting mixed class 3/4+5 component, MMF group maintained noninferiority for 24-week TRR (intention-to-treat analysis, noninferiority test, *P* = 0.007; per-protocol analysis, noninferiority test, *P* = 0.02) and CRR rates (intention-to-treat analysis, noninferiority test, *P* = 0.001; per-protocol analysis, noninferiority test, *P* = 0.002) versus the cyclophosphamide group. Pure proliferative classes (class 3 or 4) of lupus nephritis were diagnosed in 61 intention-to-treat patients and in 54 per-protocol patients. In this subgroup, the estimated difference in TRR rate between MMF group and cyclophosphamide group was −2% (95% CI, −21% to 15%; noninferiority test, *P* = 0.14) in the intention-to-treat population and −2% (95% CI, −20% to 14%; noninferiority test, *P* = 0.11) in the per-protocol population. For CRR rate, the between-group differences were −8% (95% CI, −31% to 14%; noninferiority test, *P* = 0.38) and −9% (95% CI, −32% to 13%; noninferiority test, *P* = 0.41) in intention-to-treat population and in the per-protocol population, respectively (Supplemental Tables 4 and 5).

## Discussion

Therapeutic advances have substantially improved outcomes in lupus nephritis. Since the introduction of a combination of glucocorticoids and cyclophosphamide for adult lupus nephritis in the 1980s, kidney prognosis has improved markedly.^24,25^ However, the adverse effect profile of cyclophosphamide has prompted the search for less toxic alternatives. MMF emerged in the 1990s as initial and maintenance therapy for severe lupus nephritis.^26^ Despite these advances, most clinical trials have not included pediatric patients; treatment of childhood-onset lupus nephritis is often guided by adult treatment paradigms. In this article, we report findings from a large RCT comparing the efficacy and safety of intravenous cyclophosphamide and oral MMF as initial therapy for newly diagnosed pediatric proliferative lupus nephritis with severely increased proteinuria.^27^

The management of proliferative lupus nephritis follows a biphasic approach: initial and maintenance therapy.^7,25^ Initial therapy targets remission of acute manifestations of lupus nephritis. Although several RCTs^9,10,28^ in adults with proliferative lupus nephritis have shown comparable kidney outcomes between intravenous cyclophosphamide and MMF initial regimens, a 2020 meta-analysis^29^ of 18 RCTs demonstrated superior proteinuria reduction with intravenous cyclophosphamide initial regimen for Asian patients. Pediatric data remain limited to retrospective studies. A retrospective study conducted in Turkey in 2022 analyzed 53 patients with childhood-onset lupus nephritis. Most patients received either intravenous cyclophosphamide (53.7%) or oral MMF (21.9%) as initial therapy. The study found no significant differences in TRR rates between the two treatment regimens at 6 and 12 months.^25^ Smith *et al.* retrospectively analyzed 51 juvenile patients with class 3 or 4 lupus nephritis and found no significant differences in renal British Isles Lupus Assessment Grade scores or global disease activity parameters between the intravenous cyclophosphamide and MMF groups.^30^ Similarly, in 2020, Suhlrie *et al.* reported no association between initial therapy choice (intravenous cyclophosphamide versus oral MMF) and 12-month complete remission rates in their cohort of 79 Caucasian juvenile patients with proliferative lupus nephritis.^31^ These findings were corroborated by a prospective observational study involving 41 patients with proliferative lupus nephritis showing no significant difference in renal response rates between intravenous cyclophosphamide and MMF treatment groups.^32^ The renal response outcomes in this study align with prior research, demonstrating no significant differences between the MMF and intravenous cyclophosphamide groups. MMF demonstrated noninferiority to intravenous cyclophosphamide for both TRR rates and complete renal remission at weeks 12 and 24. Furthermore, the incidence of adverse events was comparable between the MMF and intravenous cyclophosphamide groups. Gastrointestinal symptoms were surprisingly rare in our cohort. This finding is inconsistent with several studies on lupus nephritis,^9,10,31^ but there are some studies in adult^28,33^ and pediatric^34,35^ populations (not exclusively lupus nephritis) that reported similarly low rates of gastrointestinal side effects.

Nephrotic-range proteinuria was identified as a risk factor for failure to achieve complete remission.^31^ In our subgroup analysis of patients with nephrotic-range proteinuria, oral MMF demonstrated noninferiority to intravenous cyclophosphamide for renal response outcomes. Consistent with previous studies,^25,36^ class 4 lupus nephritis was the most prevalent ISN/RPS classification in our cohort. Chbihi *et al.* retrospectively analyzed 33 pediatric patients with class 4 lupus nephritis, including 17 receiving oral MMF and 16 treated with intravenous cyclophosphamide. They observed no significant differences in complete remission rates between the two treatment groups (53% versus 71%).^24^ In our class 4/4+5 lupus nephritis subgroup, MMF maintained noninferiority to cyclophosphamide for both complete and PRRs. A 2016 Kaplan–Meier analysis by Ikeuchi *et al.* demonstrated that patients with mixed class 3/4+5 lupus nephritis had significantly poorer renal outcomes than patients with class 3/4 lupus nephritis.^37^ Building on these findings, we specifically analyzed the subgroup with mixed proliferative and membranous lupus nephritis (class 3/4+5) and confirmed the noninferiority of MMF to intravenous cyclophosphamide for renal response outcomes. However, the noninferiority of the MMF group was not demonstrated at the predefined 12% noninferiority margin in the pure proliferative (class 3/4) subgroup analysis. This finding may have been affected by the limited sample size in this subgroup. Consequently, as our study was not adequately powered for these subgroup analyses, they should be interpreted as exploratory findings. Further large-scale studies are warranted to confirm these observations.

The strengths of this study include its multicenter, randomized, prospective design, which enhances the validity of the findings. In addition, by analyzing various subgroups within the proliferative lupus nephritis population, this study effectively reduced the bias associated with different subtypes. However, this study has some limitations. First, the 24-month follow-up period may be insufficient to fully evaluate long-term outcomes. A 15-year retrospective analysis by Zhang *et al.* of Chinese patients with pediatric lupus nephritis demonstrated that initial treatment with MMF was associated with a lower risk of kidney flare on multivariable analysis.^36^ However, several subsequent studies^25,30^ have shown no significant differences in the time to the first kidney flare between the MMF and intravenous cyclophosphamide groups. Notably, our study design could not assess potential long-term safety advantages of MMF regarding fertility preservation and menstrual regularity, as cyclophosphamide-related adverse effects often manifest years after treatment. We are prospectively extending follow-up to assess long-term outcomes through real-world observational study. Second, the study population consisted exclusively of Asian patients, potentially limiting the generalizability of our findings to other ethnic groups. Third, although Kidney Disease Improving Global Outcomes guidelines now recognize belimumab-based triple immunosuppressive therapy (combined with mycophenolate or cyclophosphamide plus glucocorticoids) as a potential first-line approach for proliferative lupus nephritis, our study design did not incorporate biologic agents, precluding evaluation of their potential synergistic effects. Fourth, to ensure patient safety and adhere to ethical guidelines, we excluded patients with an eGFR <60 ml/min per 1.73 m^2^, as some patients in this subgroup may experience progressive kidney function decline and require more intensive therapeutic management. This criterion necessarily restricts the generalizability of our findings.

In summary, to the best of our knowledge, this is the largest RCT conducted to date to investigate initial treatments for newly diagnosed childhood-onset proliferative lupus nephritis with severely elevated proteinuria (UPCR ≥1000 mg/g or 24-hour urinary protein excretion >25 mg/kg) across multiple centers in mainland China. The study found that oral MMF therapy was noninferior to intravenous cyclophosphamide therapy for the renal response. Based on these findings, we suggest that MMF should be considered a first-line initial therapy for childhood-onset proliferative lupus nephritis.

## Supplementary Material

## Data Availability

This study includes clinical experimentation and received Institutional Review Board or Ethics Committee approval. All patients provided written informed consent. This study includes clinical experimentation and complies with the Declaration of Helsinki.

## Data Availability Statements

Original data generated for the study are available in a repository subject to controlled access. Data Type: Aggregated Data; Clinical Trial Data. Repository Name: Figshare. Reason for Restriction: Data supporting the findings of this study are available within the article and its Supplemental Material. A hybrid dataset, comprising both deidentified participant data and derived data, is shared privately *via* Figshare and will remain available until January 1, 2027 (https://figshare.com/s/51f03e2e8ea14ba45741). Complete datasets are not shared due to a lack of informed consent from patients for the secondary use of data. Additional supporting documents, including the study protocol and statistical analysis plan, are also accessible.

## Supplemental Material

Supplemental Table 1. Baseline characteristics of the per-protocol population in a trial of pediatric proliferative lupus nephritis.

Supplemental Table 2. Primary and secondary end points at week 12 (intention-to-treat population).

Supplemental Table 3. Primary and secondary end points at week 12 (per-protocol population).

Supplemental Table 4. The renal response of different subgroups in intention-to-treat population.

Supplemental Table 5. The renal response of different subgroups in per-protocol population.

Statistical Analysis Plan

Trial Protocol
