# Supplementary material for: Mycophenolate Mofetil versus Cyclophosphamide for Initial Therapy in Childhood-Onset Proliferative Lupus Nephritis: A Prospective, Multicenter, Randomized Trial
Source: J Am Soc Nephrol. 2025 Sep 12;37(3):560–8. doi: 10.1681/ASN.0000000866 (PMC12935383; doi:10.1681/ASN.0000000866)
Supplement: Supplementary file 2 [file jasn-37-560-s002.pdf]

Supplemental Material Table of Contents

Supplemental Table 1. Baseline Characteristics of The Per-Protocol Population in a Trial of Pediatric Proliferative Lupus Nephritis
Supplemental Table 2. Primary and Secondary End Points at Week 12 (Intention-to-Treat Population)
Supplemental Table 3. Primary and Secondary End Points at Week 12 (Per-Protocol Population)
Supplemental Table 4. The Renal Response of Different Subgroups in Intention-to-Treat Population
Supplemental Table 5. The Renal Response of Different Subgroups in Per-Protocol Population

Statistical Analysis Plan

Trial Protocol

Ethical Approval Document

Ethical Approval Annual Report Review

OTHER OUTCOMES： Supplemental Tables

Supplemental Table 1. Baseline Characteristics of The Per-Protocol Population in a Trial of Pediatric Proliferative Lupus Nephritis

Characteristics	MMF Group (N = 47)	Cyclophosphamide Group (N = 48)
Age at enrollment, year, median (Q1-Q3)	12 (9-13)	12 (10-13)
Male, no. (%)	10 (21)	11 (23)
Duration of disease, day, median (Q1-Q3)	30 (12-60)	21 (10-50)
eGFR, ml/min/1.73 m^2, median (Q1–Q3)	106 (71-132)	94 (72-130)
BUN level, mg/dl, median (Q1-Q3)	18 (13-26)	22 (16-29)
Serum albumin level, g/L, mean (SD)	2.7 (0.8)	2.7 (0.7)
Hemoglobin level, g/dl, mean (SD)	10.3 (2.3)	10.1 (2.0)
24-hr urine protein, mg, median (Q1-Q3)	2970 (1414-4360)	2865 (1373-5699)
Nephrotic range proteinuria, no. (%)	33 (70)	32 (67)
Hematuria, no. (%)	37 (79)	40 (83)
Hypertension, no. (%)	20 (43)	26(54)
Kidney biopsy class, no. (%)		
III or IV	24 (51)	30 (62)
III + V or IV + V	23 (49)	18 (38)
Low C3, no. (%)	46 (98)	47 (98)
Low C4, no. (%)	41 (87)	41 (85)
dsDNA antibody positive, no. (%)	45 (96)	43 (90)
SLEDAI-2K score, mean (SD)	18 (5)	18 (5)

MMF, mycophenolate mofetil; eGFR, estimated glomerular filtration rate; BUN, blood urea nitrogen; SLEDAI-2K, Systemic Lupus Erythematosus Disease Activity Index 2000.

Data are presented as n (%), median (25-75th percentile), or mean ± standard deviation (SD).

Supplemental Table 2. Primary and Secondary End Points at Week 12 (Intention-to-Treat Population)

	MMF Group (N = 52)	Cyclophosphamide Group (N = 55)	Treatment Difference (95% CI) [a]
Total renal response, no. (%)	46 (88)	46 (84)	4 (-9 to 18)
Complete renal response, no. (%)	29 (56)	26 (47)	9 (-10 to 26)
Primary efficacy renal response, no. (%)	2 (4)	6 (11)	-7 (-18 to 4)
Partial renal response, no. (%)	15 (29)	14 (25)	4 (-13 to 20)
No renal response, no. (%)	6 (12)	9 (16)	-4 (-18 to 9)
SLEDAI-2k change from baseline, mean (SD)	11 (5)	12 (6)	-1 (-3 to 2)

MMF, mycophenolate mofetil.

[a]The treatment difference is given as rate difference or mean difference and corresponding 95% confidence intervals.

Supplemental Table 3. Primary and Secondary End Points at Week 12 (Per-Protocol Population)

	MMF Group (N = 49)	Cyclophosphamide Group (N = 52)	Treatment Difference (95%CI) [a]
Total renal response, no. (%)	45 (92)	44 (85)	7 (-6 to 20)
Complete renal response, no. (%)	29 (59)	25 (48)	11 (-8 to 29)
Primary efficacy renal response, no. (%)	2 (4)	5 (10)	-6 (-17 to 6)
Partial renal response, no. (%)	14 (29)	14 (27)	2 (-15 to 19)
No renal response, no. (%)	4 (8)	8 (15)	-7 (-20 to 6)
SLEDAI-2k change from baseline, mean (SD)	12 (5)	12 (5)	-0 (-2 to 2)

MMF, mycophenolate mofetil.

[a]The treatment difference is given as rate difference or mean difference and corresponding 95% confidence intervals.

Supplemental Table 4. The Renal Response of Different Subgroups in Intention-to-Treat Population

Subgroup	Cohort	Renal Response, no./total no. of subgroups (%)		Treatment Difference, % (95%CI)	
		Total Renal Response	Complete Renal Response	Total Renal Response	Complete Renal Response
Nephrotic range proteinuria	MMF group	33/36 (92)	27/36 (75)	7 (-8 to 23)	14(-7 to 34)
	Cyclophosphamide group	32/38 (84)	23/38 (61)		
IV or IV + V type	MMF group	40/42 (95)	33/42 (79)	2 (-10 to 15)	10 (-9 to 27)
	Cyclophosphamide group	39/42 (93)	29/42 (69)		
III + V or IV + V type	MMF group	24/24 (100)	22/24 (92)	9 (-6 to 28)	28 (4 to 50)
	Cyclophosphamide group	20/22 (91)	14/22 (64)		
III or IV type	MMF group	24/28 (86)	18/28 (64)	-2 (-21 to 15)	-8 (-31 to 14)
	Cyclophosphamide group	29/33 (88)	24/33 (73)		

MMF, mycophenolate mofetil.

Supplemental Table 5. The Renal Response of Different Subgroups in Per-Protocol Population

Subgroup	Cohort	Renal response, no./total no. of subgroups (%)		Treatment Difference, (95%CI)	
		Total Renal Response	Complete Renal Response	Total Renal Response	Complete Renal Response
Nephrotic range proteinuria	MMF group	31/33 (94)	26/33 (79)	3 (-12 to 19)	10 (-11 to 30)
	Cyclophosphamide group	29/32 (82)	22/32 (75)		
IV or IV + V type	MMF group	37/38 (97)	31/38 (82)	0 (-11 to 12)	7 (-12 to 25)
	Cyclophosphamide group	35/36 (97)	27/36 (75)		
III + V or IV + V type	MMF group	23/23 (100)	21/23 (91)	6 (-9 to 26)	25 (0 to 48)
	Cyclophosphamide group	17/18 (94)	12/18 (67)		
III or IV type	MMF group	22/24 (92)	17/24 (71)	-2 (-20 to 14)	-9 (-32 to 13)
	Cyclophosphamide group	28/30 (93)	24/30 (80)		

MMF, mycophenolate mofetil.

Mycophenolate Mofetil versus Cyclophosphamide for Induction Therapy in Childhood-Onset Proliferative Lupus Nephritis: A Prospective, Multicenter, Randomized Trial

Statistical Analysis Plan

Final version

Table of contents

1 Introduction..1

2 Design 1

2.1 Setting 1

2.2 Study Locations 2

2.3 Study population 3

2.3.1 Inclusion criteria 3

2.3.2 Exclusion criteria 4

2.4 Randomization 4

2.5 Sample size 4

3 Study schema 5

4 Primary and secondary outcomes 8

4.1 Efficacy outcomes..8

4.2 Safety outcomes..9

5. Statistical considerations..10

5.1 Descriptive Statistics..10

5.2 Type I error level..10

5.3 Analysis for primary outcomes..11

5.4 Analysis for secondary outcomes 11

5.5 Analysis for safety data..12

5.6 Interim Analysis..12

5.7 Post-hoc Analysis..12

5.8 Analysis Populations..13

5.9 Handling of Missing Data...13

6 Data analysis software 13

7 References..13

1 Introduction

Recent studies have indicated that oral mycophenolate mofetil (MMF) may be as effective as intravenous cyclophosphamide (IVC) in treating lupus nephritis (LN). However, no direct prospective comparison of these two therapies has been conducted in patients with childhood-onset LN. This current study was designed to demonstrate the noninferiority of oral MMF compared with IVC in achieving renal response at 24 weeks.

2 Design

2.1 Setting

This is an open-label, prospective, multicenter, randomized controlled trial with non-inferiority design.

The fixed-margin method was employed to establish the non-inferiority margin. As no meta-analyses have directly compared cyclophosphamide + glucocorticoid versus placebo + glucocorticoid for lupus nephritis, we referenced a randomized trial[1] that reported combination therapy (cyclophosphamide + glucocorticoid) achieved a renal remission rate of 85% (17/20) versus 29% (7/24) with glucocorticoid alone (rate difference 56%, 95% CI 27-73%). Preserving 50-80% of cyclophosphamide's treatment effect and applying the formula (1 - preservation fraction) × 27%, yielded a margin range of 5.4-13.5%. Accounting for operational challenges in patient recruitment following China's 2022 COVID-19 policy adjustments,

we conservatively selected 12% as the non-inferiority margin.

2.2 Study Locations

This study was performed at 17 institutions in China.

Table 1. Study Locations

geographical areas	institutions
North China	1. Department of Pediatrics, Peking Union Medical College Hospital, Chinese Academy of Medical Sciences, Beijing, China<br>2. Department of Immunology, Beijing Children's Hospital, Capital Medical University, National Center for Children's Health, Beijing, China<br>3. Department of Pediatrics, the Second Hospital of Hebei Medical University, Shijiazhuang, China
Northeast China	4. Department of Pediatrics, Shengjing Hospital of China Medical University, Shenyang, China
Eastern China	5. Department of Nephrology and Immunology, Children’s Hospital of Soochow University, Suzhou, China<br>6. Department of Rheumatology Immunology & Allergy; Children’s Hospital, Zhejiang University School of Medicine, Hangzhou, China<br>7. The First Affiliated Hospital of Xiamen University, Xiamen, China<br>8. Department of Nephrology, Jiangxi Provincial Children’s Hospital, Nanchang, China<br>9. Department of Pediatric Nephrology and Rheumatology and Immunology, Shandong Provincial Hospital Affiliated to Shandong First Medical University, Jinan, China<br>10. Department of pediatric nephrology and rheumatology, Affiliated Hospital of Qingdao University, Qingdao, China
Central China	11. Department of Pediatrics, the First Affiliated Hospital of Zhengzhou University, Zhengzhou, China<br>12. Department of Pediatrics, The Second Xiangya Hospital, Central South University, Changsha, China<br>13. Department of Pediatric Nephrology, Rheumatology and Immunology, the First People’s Hospital of Chenzhou, Chenzhou, China

Table 1. Study Locations (continue)

geographical areas	institutions
Southern China	14. Department of Pediatrics, Nanfang Hospital, Southern Medical University, Guangzhou, China<br>15. Department of Rheumatology and Immunology, Shenzhen Children’s hospital, Shenzhen, China
Southwest China	16. Department of Nephrology, Children’s Hospital of Chongqing Medical University, Chongqing, China<br>17. Pediatric Immunology and Rheumatology Department, Chengdu Women's and Children's Central Hospital, School of Medicine, University of Electronic Science and Technology of China, Chengdu, China

2.3 Study population

2.3.1 Inclusion criteria

1) age 5-17 years at enrollment; 2) newly diagnosed with SLE according to the 2019 American College of Rheumatology classification criteria[2] or the 2012 Systemic Lupus International Collaborating Clinics (SLICC) criteria[3]; 3) biopsy-proven proliferative lupus nephritis (class III/IV ± V) diagnosed according to the 2003 International Society for Nephrology/Renal Pathology Society (ISN/RPS) classification[4]; 4) estimated glomerular filtration rate (eGFR) $\geq$ 60 ml/min/1.73m^2, 24-hour proteinuria $\geq$ 25 mg/kg or urine protein-creatinine ratio $\geq$ 1000 mg/g, blood white blood cell count $\geq 3.0 \times 10^9$/L and lymphocyte count $\geq 0.5 \times 10^9$/L; 5) no prior use of immunosuppressants or biologics before enrollment.

2.3.2 Exclusion criteria

(1) a history of diseases predisposing to infections (e.g., primary immunodeficiency, splenectomy, or other infection-prone conditions); (2) prior diagnosis of malignance; (3) severe infections (e.g., active hepatitis C, active hepatitis B, HIV infection, active tuberculosis infection, severe fungal infection, or other serious organ infections); (4) serious complications (e.g., symptoms of central nervous system and peripheral nervous system involvement suggesting lupus encephalopathy, diffuse alveolar hemorrhage, severe hemolysis with hemoglobin less than 60 g/L, platelet count less than 10.0×10^9/L); (5) unstable vital signs (e.g., altered consciousness, arrhythmia, hypotension, hypoxia, etc.), (6) allergies to cyclophosphamide, MMF, or glucocorticoids.

2.4 Randomization

Randomization was conducted using block randomization with a block size of 6.

2.5 Sample size

Based on a previously published 24-week prospective, randomized, open-label trial comparing MMF with IVC as induction therapy for active biopsy proven Class III and IV lupus nephritis[5], we considered the total renal response (TRR) rate to be 88% in the MMF group and 87% in the IVC

group. We calculated that enrollment of approximately 190 patients up to a maximum of 224 (considering a 15% rate of patient withdrawal) would provide the trial with at least 80% power for the assessment of the primary end point within a noninferiority margin of 12.5% at a one-sided significance level of 0.025. However, The Chinese Alliance of Pediatric Rheumatic & Immunologic Diseases (CAPRID) revised the estimated total renal response rates to 84% for the MMF group and 71% for the IVC group, based on a meta-analysis of treatment outcomes in Chinese lupus nephritis patients [6]. Additionally, the noninferiority margin was adjusted to 12% at the initial stage of the study. These revisions were prompted by the relaxation of COVID-19 policies in China in 2022, which led to operational challenges in recruitment and potentially impacted the integrity of the trial data. Furthermore, the initial sample size calculation was based on a single-center study involving Indian patients. These adjustments also ensured that the sample size would be sufficient to provide adequate power for the primary endpoint measurement. Finally, the trial aimed to analyze 88 patients (44 per group) in the per-protocol population. Accounting for a 15% patient withdrawal rate, the adjusted total sample size was calculated as 104.

3 Study schema

Pediatric patients newly diagnosed with proliferative lupus nephritis in 17

different hospitals between June 2022 and May 2024 were screened for eligibility. Upon confirmation of full eligibility, patients were randomly assigned in a 1:1 ratio to receive either oral MMF plus glucocorticoids or IVC plus glucocorticoids for 6 months by the investigator.

All patients in the IVC group received cyclophosphamide therapy (750mg/m^2, not exceeding 1 g, administered over 1-2 days) monthly for 6 months combined with glucocorticoids. According to the 2016 Evidence-Based Guidelines for Diagnosis and Treatment of Steroid-Resistant Nephrotic Syndrome in China[7], the following pretreatment protocols were implemented: alkalinization therapy (Administer 5% sodium bicarbonate at 3-5 mL/kg, diluted to 1.4%, intravenously.) was administered prior to cyclophosphamide; ondansetron antiemetic was given 30 minutes before cyclophosphamide; post-cyclophosphamide hydration (total fluid tonicity maintained at 1/4-1/5 normal saline, total volume 30-50ml/kg). Notably, MESNA prophylaxis was omitted due to the exceptionally low incidence of hemorrhagic cystitis observed in Chinese pediatric nephrology practice following cyclophosphamide treatment. For patients with hypertension, volume overload, or active nephrotic syndrome, fluid restriction (<1000 mL/day) was enforced, supplemented by furosemide diuresis to maintain appropriate urine output.

All patients in the MMF group received MMF therapy (30-40 mg/kg/day, divided into two doses, not to exceeding 1g/dose) in conjunction with

glucocorticoids. The rationale for MMF dosing in mg/kg/day is as follows: The Chinese guideline for childhood SLE recommend a weight-based MMF dosage of 30-40 mg/kg/day[8]; however, previous studies[9,10] have used 20–40 mg/kg/day or a body surface area (BSA)-based dosage of 625–1299 mg/m². When converting BSA-based dosing, 625–1299 mg/m² is approximately equivalent to 25–50 mg/kg (for BSA ≤ 1) or 16–40 mg/kg (for BSA > 1). Pharmacokinetic monitoring revealed that both 960 mg/m² and 30 mg/kg doses achieve target mycophenolic acid (MPA) exposure (12-hour area under the concentration-time curve [AUC] >50 mg·h/L) in most children. Pharmacokinetic studies demonstrate that patients attaining MPA AUC >50 mg·h/L require a mean MMF dose of 30 mg/kg/day compared to 20 mg/kg/day[11]. Thus, the recommended dosage range of 30–40 mg/kg/day is justified by both clinical guidelines and pharmacodynamic data. No participants required switching to mycophenolate sodium (or other mycophenolic acid analogs) due to MMF-related adverse effects.

The CAPRID has standardized the glucocorticoids treatment therapy in accordance with the "International Consensus for The Dosing of Corticosteroids in Childhood-Onset Systemic Lupus Erythematosus with Proliferative Lupus Nephritis"[12] (Table 2 showed detailed information on glucocorticoids dosing and tapering schedules). All patients were given supportive therapy including the hydroxychloroquine (4-6 mg/kg/day), angiotensin-converting enzyme inhibitors.

Table 2 Standardized prednisone dosing regimen.

time	Weight ≥40kg	Weight 30~<40kg	Weight 25~<30kg	Weight 20~<25kg
Week 1	60 mg#	60 mg#	50 mg#	40 mg#
Week 2	60 mg#	60 mg#	50 mg#	40 mg#
Week 3	50 mg	50 mg	40 mg	35 mg
Week 4	50 mg	50 mg	40 mg	35 mg
Week 5-6	40 mg	40 mg	35 mg	30 mg
Week 7-8	40 mg	40 mg	35 mg	30 mg
Week 9-10	35 mg	30 mg	30 mg	25 mg
Week 11-12	35 mg	30 mg	30 mg	25 mg
Week 13-14	30 mg	25 mg	20 mg	20 mg
Week 15-16	30 mg	25 mg	20 mg	20 mg
Week 17-18	25 mg	20 mg	15 mg	15 mg
Week 19-20	25 mg	20 mg	15 mg	15 mg
Week 21-22	20 mg	15 mg	10 mg	10 mg
Week 23-24	20 mg	15 mg	10 mg	10 mg

#Patients were administered high-dose methylprednisolone infusions (15–30 mg/kg per dose, with a maximum of 500 mg per day) three times per week for two consecutive weeks following the diagnosis of proliferative LN.

4 Primary and secondary outcomes

All participants received regular follow-up assessments with monitoring intervals adjusted according to therapeutic response, wherein patients with incomplete renal response underwent evaluation every 4 weeks, whereas complete responders were assessed at baseline followed by subsequent evaluations at 4, 8, 12, and 24 weeks.

4.1 Efficacy outcomes

The primary endpoint was the total proportion of patients who achieved a renal response to treatment at 24-week milestones, defined as the

composite of complete renal response (CRR), primary efficacy renal response (PERR), and partial renal response (PRR). The definitions of treatment response in lupus nephritis were based on the Kidney Disease: Improving Global Outcomes (KDIGO) 2024 Clinical Practice Guideline for the Management of LN [13]. Complete renal response is defined as proteinuria < 0.5g/1.73 m^2 per day or <300mg/m^2 per day based on a 24-hour urine specimen, along with stabilization or improvement in kidney function (±10%-15% of baseline). Primary efficacy renal response was defined as a protein-creatinine ratio (PCR) < 700 mg/g and an estimated glomerular filtration rate (eGFR) no worse than 20% below the pre-flare value or ≥ 60ml/min per 1.73 m^2. Partial renal response was defined as a reduction in proteinuria by at least 50%, PCR < 3000 mg/g and stabilization or improvement in kidney function (±10%-15% of baseline). No renal response (NRR) was defined as failure to achieve either a partial or complete renal response. The change in the SLEDAI-2K score from baseline is one of key secondary endpoints.

4.2 Safety outcomes

Adverse event incidence was a secondary endpoint. Study withdrawal may be considered for adverse events (not related to the disease under investigation) that reach Common Terminology Criteria for Adverse Events (CTCAE) Grade 3 or higher severity. Cases meeting the ICH E6(R3)

Good Clinical Practice definition of serious adverse events (SAEs) must be reported to the sponsor immediately, while adverse events (AEs) should be submitted as follow-up reports. Notably, any patient who withdrew from the study due to disease-related adverse events—including (1) renal flare, (2) persistent extrarenal manifestations, or (3) failure to achieve partial renal response by week 12—was considered to have experienced an SAE, except for cases of adverse drug reactions. The assessment of adverse drug reactions included infections, menstrual disorders, hematologic abnormalities, and other adverse events. According to the WHO-UMC causality assessment system for evaluating the causal relationship between medicine and AEs.

5. Statistical considerations

5.1 Descriptive Statistics

Continuous variables are presented as mean ± standard deviation (SD) if normally distributed, or as median (P25, P75) if not normally distributed. Categorical variables are described using number (percentage). The differences in renal response rates and SLEDAI - 2K scores between the two groups are presented as differences in rates or mean values.

5.2 Type I error level

For the non - inferiority test, the significance level (α) is set at 0.025 (one

- sided). For the difference test, a two - sided approach is adopted with a significance level of 0.05.

5.3 Analysis for primary outcomes

The two-side 95% confidence intervals (CIs) for renal response rate differences (RDs) were calculated using the Newcombe-Wilson method (Figure 1). Noninferiority analyses of both the total renal response and complete renal response rates between the two groups were performed using Farrington-Manning (FM) tests, with a prespecified noninferiority margin of 12%.

$$\frac{2np + z^2 \pm z\sqrt{z^2 + 4npq}}{2(n + z^2)}$$

$$L = p_1 - p_2 - \sqrt{(p_1 - l_1)^2 + (u_2 - p_2)^2}$$

$$U = p_1 - p_2 - \sqrt{(p_2 - l_2)^2 + (u_1 - p_1)^2}$$

Figure 1. Newcombe-Wilson method

Where, q=1-p and z=1.96, at the same time, the upper and lower limits for rates of the two groups (p_1 and p_2), l_1, u_1, l_2, u_2, can be calculated by the first formula. Then, the confidence interval of the rate difference between the two groups can be calculated according to the formula.

5.4 Analysis for secondary outcomes

The mean difference in SLEDAI-2K score and its two-sided 95% confidence interval (CI) were calculated using the t-test.

5.5 Analysis for safety data

Categorical variables related to adverse drug effects were analyzed using the chi-squared test.

5.6 Interim Analysis

When all enrolled patients completed the 3-month follow-up, data collected during this period were used for the interim analysis.

5.7 Post-hoc Analysis

We conducted a post-hoc subgroup noninferiority analysis of renal response across difference subgroups at week 24: Lupus nephritis with nephrotic-range proteinuria, class IV lupus nephritis, proliferative with membranous lupus nephritis, pure proliferative lupus nephritis. The subgroup definitions were as follows: (1) Lupus nephritis with nephrotic-range proteinuria[14]: 24-hour urinary protein excretion >50 mg/kg or urine protein-to-creatinine ratio >2000 mg/g; (2) Class IV lupus nephritis: Kidney biopsy showing ISN/RPS Class IV or IV+V; (3) Proliferative with membranous lupus nephritis: Kidney biopsy demonstrating ISN/RPS Class III+V or IV+V. (4) Pure proliferative lupus nephritis: Kidney biopsy demonstrating ISN/RPS Class III or IV.

5.8 Analysis Populations

The intention-to-treat (ITT) population included all patients whose eligibility was confirmed and who underwent randomization. The per-protocol (PP) population consisted of eligible randomized patients who completed the 24-week treatment according to original allocation. For the 12-week interim analysis, the PP population included eligible randomized patients who completed the 12-week treatment per protocol.

5.9 Handling of Missing Data

For patients who dropped out, the last available data were used for intention-to-treat analysis.

6 Data analysis software

Statistical analysis was performed using Statistical Analysis System software (version 9.4; SAS Institute, Cary, NC) and SPSS Statistics (version 26.0; IBM Corp., Armonk, NY, United States).

7 References

[1] Gourley MF, Austin HA 3rd, Scott D, et al. Methylprednisolone and cyclophosphamide, alone or in combination, in patients with lupus nephritis. A randomized, controlled trial. *Ann Intern Med*. 1996;125(7):549-557. doi:10.7326/0003-4819-125-7-199610010-00003

[2] Aringer M, Costenbader K, Daikh D, et al. 2019 European League Against Rheumatism/American College of Rheumatology Classification Criteria for Systemic Lupus Erythematosus. *Arthritis Rheumatol*. 2019;71(9):1400-1412. doi:10.1002/art.40930

[3] Petri M, Orbai AM, Alarcón GS, et al. Derivation and validation of the Systemic Lupus International Collaborating Clinics classification criteria for systemic lupus erythematosus. *Arthritis Rheum*. 2012;64(8):2677-2686. doi:10.1002/art.34473

[4] Weening JJ, D'Agati VD, Schwartz MM, et al. The classification of glomerulonephritis in systemic lupus erythematosus revisited. *J Am Soc Nephrol*. 2004;15(2):241-250. doi:10.1097/01.asn.0000108969.21691.5d

[5] Mendonca S, Gupta D, Ali S, Gupta P. Mycophenolate mofetil or cyclophosphamide in indian patients with lupus nephritis: Which is better? A single-center experience. S*audi J Kidney Dis Transpl*. 2017;28(5):1069-1077. doi:10.4103/1319-2442.215147

[6] Zhang H, Zhou M, Han X, et al. Mycophenolate mofetil in the treatment of Chinese patients with lupus nephritis: A PRISMA-compliant meta-analysis. *Medicine (Baltimore)*. 2020;99(33):e21121.doi:10.1097/MD.0000000000021121

[7] Subspecialty Group of Nephrology, Society of Pediatrics, Chinese Medical Association. Evidence-Based Guidelines for Diagnosis and Treatment of Steroid-Resistant Nephrotic Syndrome. *Zhonghua Er Ke Za Zhi*, 2017,55(11):805-809. DOI:10.3760/cma.j.issn.0578-1310.2017.11.002.

[8] Subspecialty Group of Immunology, the Society of Pediatrics, Chinese Medical Association; Editorial Board, Chinese Journal of Pediatrics. Chinese guidelines for the diagnosis and treatment of childhood-onset systemic lupus erythematosus. *Zhonghua Er Ke Za Zhi*. 2021;59(12):1009-1024. doi:10.3760/cma.j.cn112140-20210905-00743

[9] Li Z, Wang J, Huang Y, et al. Identification of biomarkers that predict renal flare in childhood-onset lupus nephritis with mycophenolate acid.

Int Immunopharmacol. 2023;117:109900. doi:10.1016/j.intimp. 2023.109900

[10] Aragon E, Chan YH, Ng KH, et al. Good outcomes with mycophenolate-cyclosporine-based induction protocol in children with severe proliferative lupus nephritis. Lupus. 2010;19(8):965-973. doi:10.1177/0961203310366855

[11] Sharma AP, Medeiros M, Norozi S, et al. Clinical applicability of 2023 International Pediatric Nephrology Association recommended limited therapeutic drug monitoring formulae to assess mycophenolic acid exposure. *Pediatr Nephrol*. 2025;40(6):1965-1973. doi:10.1007/ s00467-025-06657-w

[12] Chalhoub NE, Wenderfer SE, Levy DM, et al. International Consensus for the Dosing of Corticosteroids in Childhood-Onset Systemic Lupus Erythematosus with Proliferative Lupus Nephritis. *Arthritis Rheumatol*. 2022;74(2):263-273. doi:10.1002/art.41930

[13] Kidney Disease: Improving Global Outcomes (KDIGO) Lupus Nephritis Work Group. KDIGO 2024 Clinical Practice Guideline for the management of LUPUS NEPHRITIS. *Kidney Int*. 2024;105(1S): S1-S69. doi:10.1016/j.kint.2023.09.002

[14] Subspecialty Group of Renal Diseases, the Society of Pediatrics, Chinese Medical Association. Evidence-based guideline on diagnosis and treatment of steroid-sensitive, relapsing/steroid-dependent nephrotic syndrome in children(2016). Zhonghua Er Ke Za Zhi. 2017;55(10):729-734. doi:10.3760/cma.j.issn.0578-1310.2017.10.003

Mycophenolate Mofetil Versus Cyclophosphamide in the Induction Therapy of Pediatric Active Proliferative Lupus Nephritis: A Prospective, Multicenter, Randomized Study (MyCITS)

**Main research unit:**	The Second Xiangya Hospital, Central South University
**Principal investigator：**	Xiaochuan Wu
**Sponsor:**	National Key Research and Development Program of China (2021YFC2702004)
**Study Design：**	Prospective multicenter study
**Participation Role:**	Lead institution
**Version No.:**	V 2.1
**Version date:**	December 10, 2022

This clinical trial protocol is confidential and owned by the Sponsor. Without the permission from the Sponsor, it is not allowed to provide the contents, in part or in full, to any third party (group or individual).

Contents

Compliance Statement **1**

For Investigator's Signature **2**

Abbreviations **3**

Protocol Abstract **5**

I Abstract 5
II Research Plan 7
Ⅲ Clinical Trial Flow Chart 8
IV Study Sites 10

1. Background **11**

2. Purpose **13**

2.1 To observe the efficacy of test drugs 13
2.2 To observe the safety outcomes of test drugs 13

3. Design basis, type, principle and case allocation **13**

3.1 Design basis 13
3.2 Design type 13
3.3 Design principle and case allocation 13
3.3.1 Noninferiority analysis 13
3.3.2 Determination of the number of cases and case allocation 14

4. Selection of cases **14**

4.1 Inclusion criteria 14
4.2 Exclusion criteria 15
4.3 Elimination criteria 15
4.4 Withdrawal criteria 15
4.5 Drop-out and treatment 16
4.5.1 Definition of drop-out 16
4.5.2 Treatment of drop-off cases: 17

5. Test drugs and their allocation **17**

5.1 Drug name and specifications 17
5.2 Administration method and dose 17
5.3 Concomitant medication 19

6. Test process and steps **20**

7. Evaluation of efficacy and safety **20**

7.1 Efficacy outcomes 20

7.2 Efficacy criteria....20
7.3 Safety evaluation....21
7.4 Exploratory Outcomes:....21

8. Reporting adverse events....21

8.1 Definition of adverse events....21
8.1.1 Concerned adverse reactions of test drug MMF....22
8.1.2 Concerned adverse reactions of test drug cyclophosphamide....23
8.2 Causality assessment....24
8.3 Records of adverse events....25
8.3.1 Components of adverse event data collection....25
8.3.2 Cycle of adverse event collection....25
8.3.3 Follow-up of unresolved adverse events....26
8.3.4 Variables....26
8.4.5 Adverse events based on signs and symptoms....27
8.4 Report of SAEs....28

9. Quality control and assurance of the trial....29

9.1 Quality control measures in laboratory....29
9.2 Quality control and assurance in the trial process....29
9.3 Measures to improve subject compliance....30
9.4 Quality control and quality assurance systems....30

10. Data management....30

11. Statistical analysis....31

11.1 Analysis data set....31
11.2 Statistical method....31
11.2.1 Descriptive statistics....31
11.2.2 Between-group difference values....31
11.2.3 Type I error level....32
11.2.4 Non-inferiority margin....32
11.2.5 Handling of Missing Data....32
11.2.6 Interim Analysis....32
11.2.7 Primary efficacy analysis....32
11.2.8 Secondary efficacy analysis....32
11.2.9 Safety analysis....33
11.3 Data analysis software....33

12. Ethical principles....33

12.1 Ethical review....33
12.2 Benefits and risks....33
12.3 Informed consent process....33
12.4 Medical treatment and protection of subjects....34

12.5 Protection of subject privacy 34

13. Ethical requirements...34

14. Data preservation..34

15. Follow-up and medical measures after the trial...35

16. References..35

Appendix..38

Appendix 1 2019 American College of Rheumatology classification criteria for SLE 38

Appendix 2 2012 Systemic Lupus International Collaborating Clinics criteria......39

Appendix 3 classification of lupus nephritis according to the 2003 ISN/RPS; 41

Appendix 4 SLEDAI-2K 42

Appendix 5 Estimation of glomerular filtration rate..43

Appendix 6 Parameters and results sample size calculation....................................43

Compliance Statement

This clinical research will comply with the Good Clinical Practice (GCP) and relevant regulations of the China Food and Drug Administration (CFDA).

The investigators and clinical research staff responsible for the implementation, management and supervision of this project need to complete GCP training.

This Protocol, Informed Consent Form, recruitment materials and other subject materials have been

Submitted to the Ethics Committee for review and approval. Subject recruitment would begin after the Protocol and Informed Consent Form are approved by the Ethics Committee. In addition, all revisions to the Informed Consent Form shall be approved by the Ethics Committee. Subjects who have signed the old Informed Consent Form and are still in the groups shall resign the new version of Informed Consent Form.

For Investigator's Signature

I hereby agree:

- To be responsible for the normal operation of this research in our center.
- To carry out this research in accordance with the research protocol and its amendment as well as the relevant research implementation specifications provided.
- Not to change or violate this research protocol without the review and written approval from the Sponsor and the Medical Ethics Committee, unless it is for the purpose to eliminate the harm to the subjects as soon as possible or for needs of trial management (requiring permission from the corresponding regulatory authority).
- I am fully familiar with the rational application of the research medicine described in the Protocol.
- I understand and will abide by the GCP and all relevant regulations and rules.
- To ensure that all personnel assisting me in this research are fully familiar with the research medicine as well as the responsibilities and functions related to this research as described in this Protocol.
- To ensure that there are no financial relationships between the researchers and the pharmaceutical production company.

Research center: The Second Xiangya Hospital, Central South University

Investigator (signature): Wu Xiaochuan

Date: December 28, 2022

Abbreviations

Abbreviations	Description
MMF	Mycophenolate mofetil
IVC	Intravenous cyclophosphamide
CYC	Cyclophosphamide
SLE	Systemic lupus erythematosus
LN	Lupus nephritis
ISN/RPS	International Society of Nephrology/Renal Pathology Society
SLICC	Systemic Lupus International Collaborating Clinics
UPCR	urine protein-creatinine ratio
ALT	Alanine aminotransferase
ALB	Albumin
TBiL	Total bilirubin
eGFR	Estimated glomerular filtration rate
SLEDAI-2K	SLE disease activity index-2000
TRR	Total renal response
CR	Complete remission
CRR	Complete renal response
PERR	Primary efficacy renal response
PRR	Partial renal response
NRR	No renal response
CRF	Case report form
CTCAE	Common terminology criteria for adverse events
ICH	International Council for Harmonisation
HIV	Human immunodeficiency virus
COVID	COronaVIrus disease
AE	Adverse event
SAE	Serious adverse event
DAE	Drug adverse event
WBC	White blood cell
ITT	Intention-to-treat
PP	Per-protocol

Abbreviations	Description
MD	Mean difference
SD	Standard deviation
RD	Rate difference
CI	Confidence interval
eCRF	Electronic case report form
GCP	Good clinical practice

Protocol Abstract

I Abstract

Title: Mycophenolate Mofetil Versus Cyclophosphamide in the Induction Therapy of Pediatric Active Proliferative Lupus Nephritis: A Prospective, Multicenter, Randomized Study

Objective: To compared the efficacy and safety of oral mycophenolate mofetil (MMF) plus glucocorticoids versus intravenous cyclophosphamide (IVC) plus glucocorticoids as induction therapy for renal remission in pediatric patients with proliferative lupus nephritis.

Design: Prospective, open-label, multicenter, randomized, noninferiority study.

Primary center: The Second Xiangya Hospital, Central South University

Test drug name: Mycophenolate Mofetil Dispersible; Cyclophosphamide. The choice of drug manufacturer depends on the specific drugs used by individual hospitals.

Subjects: Newly diagnosed pediatric patients with proliferative lupus nephritis, requiring eGFR ⩾ 60 ml/min/1.73m^2 and either urine protein-creatinine ratio (UPCR) ⩾ 1000 mg/g or 24-hour proteinuria > 25 mg/kg.

Treatment course: 24 weeks.

Medication:

(1) Mycophenolate mofetil group: Mycophenolate mofetil, tablets, 30-40mg/kg/day, divided into two doses, the maximum amount is no more than 2g/d.

(2) Cyclophosphamide group: Cyclophosphamide for injection, 750 mg/m^2 each month, administered over 1–2 days, not exceeding 1 g/month, once a month for 6 consecutive months.

(3) Glucocorticoids dose: Intravenous methylprednisolone pulse therapy should be administered at 15–30 mg/kg/day (maximum 500 mg/day) for three consecutive days weekly over two weeks. Following the pulse therapy interval and completion of the pulse therapy regimen, treatment should transition to oral prednisone.

Efficacy outcomes:

Primary observation outcomes: Renal response rate at 24 weeks (total renal response rate and complete renal response rate)

Secondary observation outcomes: Changes in disease activity index.

Exploratory Outcomes: Treatment response at 12 weeks, renal response of different subgroups (participants with nephrotic-range proteinuria, class IV or IV+V lupus nephritis, class III or III+V lupus nephritis, class III+V or IV+V lupus nephritis, class III or IV lupus nephritis)

Safety outcomes: Primary disease-related adverse events (inadequate treatment response or lupus flare), adverse events with the study medication.

Sample size: 88 cases. Including 44 cases in MMF Group, and 44 cases in IVC Group.

Statistical analysis: SAS 9.4 software for data statistical analysis.

II Research Plan

Research Plan

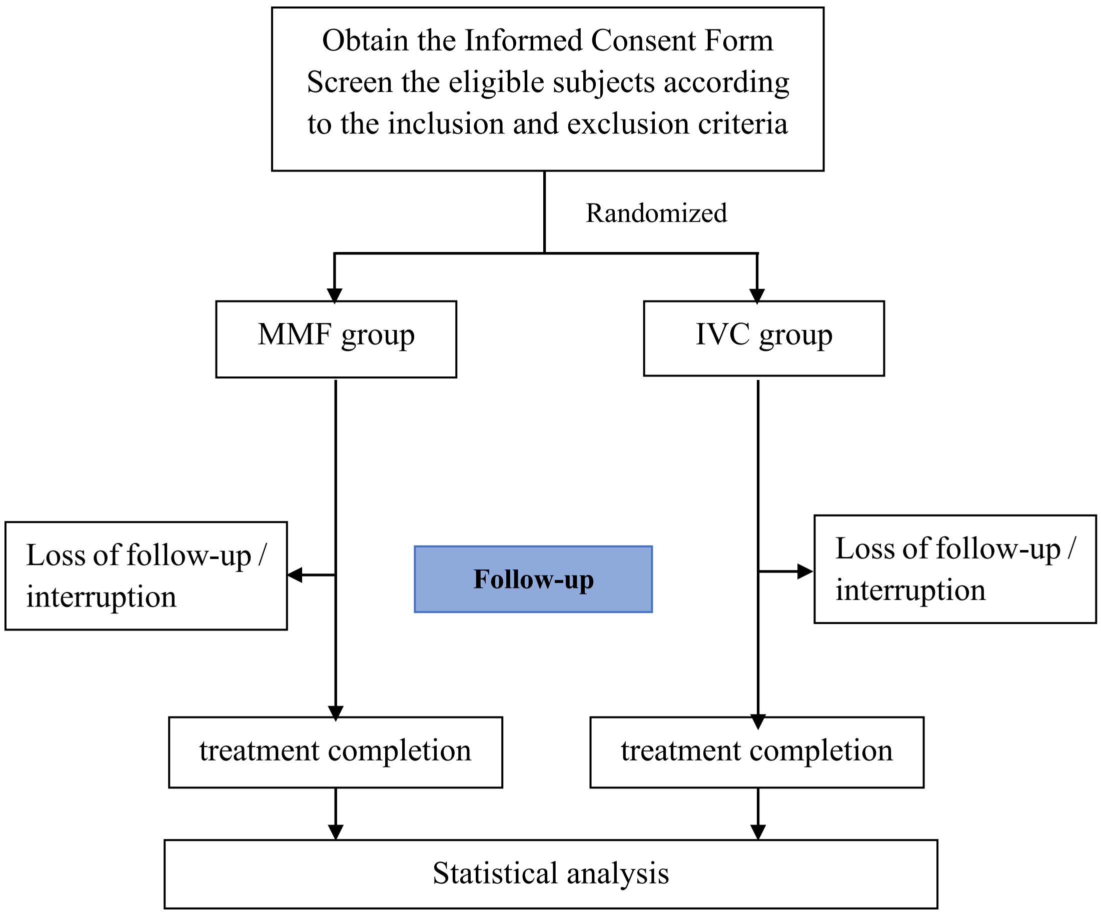

III Clinical Trial Flow Chart

Clinical trial flow chart

Items	Screening and enrollment period		Treatment and follow-up period			
	Screen	Visit 1	Visit 2	Visit 3	Visit 4	Visit 5
Follow-up week	Day -15 to 0	0 Included	4 weeks (28±7)	8 weeks (56±7)	12 weeks (84±7)	24 weeks (168±7)
Sign the Informed Consent Form	√					
Inclusion/Exclusion Criteria		√				
Randomized		√				
General data	√					
Medical history (course of disease)	√					
Vital signs	√	√	√	√	√	√
Height and weight	√	√	√	√	√	√
Physical examination	√	√	√	√	√	√
Menstrual status	√	√	√	√	√	√
Blood routine examination	√		√	√	√	√
Serum liver function	√		√	√	√	√
Serum renal function	√		√	√	√	√
Fasting blood glucose	√		√	√	√	√
Urine routine examination	√		√	√	√	√
Erythrocytes in urine	√		√	√	√	√
24h urinary protein	√		√	√	√	√
Urinary protein / creatinine	√		√	√	√	√
Kidney pathology grading	√					
ANA	√		√	√	√	√
ds-DNA	√		√	√	√	√
C3	√		√	√	√	√
C4	√		√	√	√	√
eGFR	√		√	√	√	√
Examination of mycobacterium tuberculosis and fungi	√					
HIV	√					
Markers of hepatitis B and hepatitis C virus	√					
Chest X-ray	√					

ECG	√					
SLEDAI-2000	√	√	√	√	√	√
Concomitant medication	√	√	√	√	√	√
Extrarenal clinical manifestations			√	√	√	√
Adverse event			√	√	√	√
Record case report form	√	√	√	√	√	√

Remarks:

1. Liver function: ALT, TBiL, ALB; Renal function: creatinine, urea nitrogen, uric acid;

2. Urinary protein / creatinine: morning urine; Markers of hepatitis B and hepatitis C virus: HBsAg, HBeAg, HBcAb, HCV;

3. Mycobacterium tuberculosis examination: T-sport; Fungal examination: fungal G test or GM test; Estimated glomerular filtration rate: eGFR = 0.413 × height (cm) / serum creatinine (mg /dL)；

4. This research accepts the examination data from our hospital within 2 weeks before the screening period, and no repeated test is needed.

5. Kidney pathology grading followed the 2003 International Society of Nephrology/Renal Pathology Society (ISN/RPS) lupus nephritis classification system.

IV Study Sites

(1) Department of Pediatrics, Peking Union Medical College Hospital, Chinese Academy of Medical Sciences, Beijing, China
(2) Department of Immunology, Beijing Children's Hospital, Capital Medical University, National Center for Children's Health, Beijing, China
(3) Department of Pediatrics, the Second Hospital of Hebei Medical University, Shijiazhuang, China
(4) Department of Pediatrics, Shengjing Hospital of China Medical University, Shenyang, China
(5) Department of Nephrology and Immunology, Children’s Hospital of Soochow University, Suzhou, China
(6) Department of Rheumatology Immunology & Allergy; Children’s Hospital, Zhejiang University School of Medicine, Hangzhou, China
(7) The First Affiliated Hospital of Xiamen University, Xiamen, China
(8) Department of Nephrology, Jiangxi Provincial Children’s Hospital, Nanchang, China
(9) Department of Pediatric Nephrology and Rheumatology and Immunology, Shandong Provincial Hospital Affiliated to Shandong First Medical University, Jinan, China
(10) Department of pediatric nephrology and rheumatology, Affiliated Hospital of Qingdao University, Qingdao, China
(11) Department of Pediatrics, the First Affiliated Hospital of Zhengzhou University, Zhengzhou, China
(12) Department of Pediatrics, The Second Xiangya Hospital, Central South University, Changsha, China
(13) Department of Pediatric Nephrology, Rheumatology and Immunology, the First People’s Hospital of Chenzhou, Chenzhou, China
(14) Department of Pediatrics, Nanfang Hospital, Southern Medical University, Guangzhou, China
(15) Department of Rheumatology and Immunology, Shenzhen Children’s hospital, Shenzhen, China
(16) Department of Nephrology, Children’s Hospital of Chongqing Medical University, Chongqing, China
(17) Pediatric Immunology and Rheumatology Department, Chengdu Women's and Children's Central Hospital, School of Medicine, University of Electronic Science and Technology of China, Chengdu, China

1. Background

Systemic lupus erythematosus (SLE) is a chronic autoimmune disease characterized by the presence of nuclear autoantibodies and inflammation of multiple organs [1]. Lupus nephritis (LN), one of the most severe organ manifestations, occurs in about 20-70% of SLE patients [2,3]. Childhood onset systemic lupus erythematosus (cSLE), defined as onset before 18 years of age, not only presents a higher proportion of cases developing lupus nephritis compared to adult-onset SLE but also tends to manifest more severe disease in pediatric populations [4,5].

Proliferative lupus nephritis, classified as International Society of Nephrology/Renal Pathology Society (ISN/RPS) [6] class III, IV, III+V, or IV+V, is typically more aggressive and may lead to acute kidney injury, potentially resulting in irreversible nephron loss and premature death without effective treatment [3]. The management of proliferative lupus nephritis follows a biphasic approach consisting of induction and maintenance therapy. Current Chinese guidelines demonstrate evolving recommendations for induction therapy: while the 2016 pediatric guidelines[7] recommend a 6-month induction regimen prioritizing glucocorticoids combined with intravenous cyclophosphamide (CYC), with mycophenolate mofetil (MMF) as an alternative, the 2019 guidelines[8] for adult patients expand treatment options to include MMF, CYC, or multi-target therapy as initial induction regimens for proliferative lupus nephritis (classes III/IV), with or without coexisting class V membranous features. In 2005, The New England Journal of Medicine published a 24-week, randomized, open-label, noninferiority trial in patients with class III, IV, or V lupus nephritis, demonstrating that MMF was superior to intravenous cyclophosphamide (IV CYC) in inducing remission[9]. However, a 2009 multinational, two-phase (induction and maintenance) study focusing on class III through V lupus nephritis found no significant difference in response rates between MMF and IV CYC[10]. Additionally, a meta-analysis of 14 Chinese clinical studies revealed that MMF achieved a higher complete response rate than CYC[11]. In 2017, the Single Hub and Access Point for Pediatric

Rheumatology in Europe (SHARE)[12] initiative recommended MMF or IV CYC combined with glucocorticoids as the initial regimen for proliferative lupus nephritis in pediatric patients, a recommendation largely based on clinical trials in adults and few retrospective observational studies in children[13].

To date, no adequately powered randomized controlled trials (RCTs) have directly compared induction regimens for childhood-onset lupus nephritis. Therefore, the purpose of our study is to compare the efficacy and safety of oral MMF plus glucocorticoids versus IV CYC plus glucocorticoids as induction therapy for renal remission in newly diagnosed pediatric patients with proliferative lupus nephritis who have either a urine protein-creatinine ratio (UPCR) ≥1000 mg/g or 24-hour urinary protein excretion >25 mg/kg, through a multicenter, open-label, randomized, noninferiority study.

2. Purpose

2.1 To observe the efficacy of test drugs

Primary observation outcomes: Total proportion of patients who achieved a renal response.

Secondary observation outcomes: The change of SLE activity.

2.2 To observe the safety outcomes of test drugs

Changes of vital signs (respiration and heart rate), routine blood and urine tests, liver function, renal function, blood glucose, menstrual status, and observation of clinically reported adverse events and adverse reactions.

3. Design basis, type, principle and case allocation

3.1 Design basis

(1) Drug Administration Law of the People's Republic of China (2001)

(2) Drug Registration Regulation (2007)

(3) Declaration of Helsinki (2008)

(4) Results of pharmacodynamic and toxicological studies of mycophenolate mofetil and cyclophosphamide ingredients, and current clinical trial experience

3.2 Design type

This trial adopts a multicenter, prospective, randomized, open-label interventional clinical research method. The whole observation period is 24 weeks for this trial (See III “Clinical trial flow chart” for details).

3.3 Design principle and case allocation

3.3.1 Noninferiority analysis

In this research, Farrington-Manning testing is used to compare the total renal response and complete renal response rates between the two groups.

3.3.2 Determination of the number of cases and case allocation

Eligible patients will be randomly assigned to either the MMF Group or the IVC Group. Randomization will be performed using a computer-generated random number list with a 1:1 allocation ratio and a block size of 6. Allocation will be concealed using sequentially numbered, sealed, opaque envelopes. The corresponding randomization envelope may only be opened at the time of group assignment. Any random number that is dislodged during the process cannot be reused.

Based on a published meta-analysis focusing on the treatment outcomes of Chinese patients with lupus nephritis[11], we considered the total renal response (TRR) rate (complete + primary efficacy + partial responses) to be 84% in the MMF group and 71% in the IVC group. The enrollment time of this research is expected to be 2 years, and the total time of this research is 3 years, with one-sided type I error of 0.025, 80% power, and a noninferiority margin of 12%. It is estimated to have 44 patients in MMF group and 44 patients in IVC group, with a total of 88 patients (see Appendix 6 for details). With the consideration of 15% loss of follow-up, 104 patients are needed, including 52 patients in each group.

4. Selection of cases

4.1 Inclusion criteria

Only those who meet all the conditions listed in this column can be considered for inclusion in this research:

(1) Age 5-17 years at enrollment;

(2) Newly diagnosed with SLE according to the 2019 American College of Rheumatology classification criteria[14] or the 2012 Systemic Lupus International Collaborating Clinics (SLICC) criteria[15];

(3) Biopsy-proven proliferative lupus nephritis (class III/IV ± V) diagnosed according to the 2003 ISN/RPS[6];

(4) Estimated glomerular filtration rate (eGFR) ≥ 60 ml/min/1.73m^2, 24-hour

proteinuria ≥ 25 mg/kg or urine protein-creatinine ratio ≥ 1000 mg/g, blood white blood cell count ≥ 3.0×10^9/L and lymphocyte count ≥ 0.5×10^9/L;

(5) No prior use of immunosuppressants or biologics before enrollment.

4.2 Exclusion criteria

Anyone with one of the following conditions cannot be included in this research:

(1) A history of diseases predisposing to infections (e.g., primary immunodeficiency, splenectomy, or other infection-prone conditions);

(2) Prior diagnosis of malignance;

(3) Severe infections (e.g., active hepatitis C, active hepatitis B, HIV infection, active tuberculosis infection, severe fungal infection, or other serious organ infections);

(4) Serious complications (e.g., symptoms of central nervous system and peripheral nervous system involvement suggesting lupus encephalopathy, diffuse alveolar hemorrhage, severe hemolysis with hemoglobin less than 60 g/L, platelet count less than 10.0×10^9/L);

(5) Unstable vital signs (e.g., altered consciousness, arrhythmia, hypotension, hypoxia, etc.);

(6) Allergies to cyclophosphamide, MMF, or glucocorticoids.

4.3 Elimination criteria

(1) Those who fail to take the test drugs as required, with a compliance <80% or > 120 %;

(2) Those who cannot cooperate well or complete the follow-up as planned, and those who change the drug dose at will;

4.4 Withdrawal criteria

Patients are free to withdraw from the trial at any time without any reason.

Patients must withdraw from this research in the following conditions:

(1) Withdrawal of the Informed Consent Form;

(2) No administration of the test drug according to the Protocol for 14 consecutive days due to various reasons;

(3) For two consecutive times, the measured value of serum creatinine with an interval of at least 4 weeks has increased more than twice the baseline, and eGFR < 60 $mL/min.1.73m^2$;

(4) The subject shows aggravated condition or has other symptoms affecting the trial observation during the trial, and the clinician believes that he/she should withdraw from the clinical trial;

(5) ALT or total bilirubin levels reach more than 3 times of the upper limit after medication, with a continuous increase for 2 weeks; if ALT or total bilirubin levels still rise to more than 2 times of the upper limit after 2 weeks of liver protection drug treatment, stop the test drug. In case of no recovery after 2 weeks of drug withdrawal, the patient shall withdraw from the test;

(6) Study withdrawal may be considered for adverse events (not related to the disease under investigation) that reach Common Terminology Criteria for Adverse Events (CTCAE) Grade 3 or higher severity.

(7) Occurrence of unknown adverse events of unacceptable nature, severity and/or duration, or occurrence of known unacceptable adverse events or the occurrence frequency exceeding expectations;

(8) The clinical events or safety-affecting events result in withdrawal from the clinical trial, and the investigators believe that such subjects shall withdraw from the trial;

(9) Unauthorized use of drugs prohibited by this research (see "5.3 Concomitant medication");

4.5 Drop-out and treatment

4.5.1 Definition of drop-out

All patients who have completed the Informed Consent Form and screened to be enrolled in the trial have the right to withdraw at any time. Whenever and for whatever reason, subjects who fail to complete the observation period specified in the Protocol

are considered as drop-out cases.

4.5.2 Treatment of drop-off cases:

The investigators shall contact the drop-off subjects by means of visiting, making an appointment for follow-up, calling, letter and so on, to ask the reasons, record the last medication time and complete the evaluation items that can be completed. For the cases withdrawing from the trial due to allergy or other adverse reactions and ineffective treatment, the investigators shall take corresponding treatment measures according to the actual situation of the subjects. The investigators shall properly keep the relevant test data of the drop-off cases, not only for filing, but also for full analysis and statistics.

5. Test drugs and their allocation

5.1 Drug name and specifications

(1) Mycophenolate mofetil dispersible tablets (tablets, 250 mg/tablet); Storage conditions: Store under 30°C and keep away from sunlight.

(2) Cyclophosphamide for Injection (injection, 0.2g/vial), Storage conditions: Store at a temperature not exceeding 25°C. The prepared solution must be used within 24 hours (should be stored below 8°C).

(3) Prednisone tablets (tablets, 5 mg/tablet): Storage conditions: Keep away from sunlight, and store at room temperature after sealing.

5.2 Administration method and dose

(1) MMF dose: Oral administration. 30–40 mg/kg/day, divided into two doses, not to exceed 1 g/dose;

(2) Cyclophosphamide dose: Intravenous infusion. 750 mg/m²/month, administered over 1–2 days, not exceeding 1 g/month.

[1) The pretreatment protocol included intravenous 1.4% sodium bicarbonate (3-5 ml/kg) before cyclophosphamide, ondansetron antiemetic administered 30 minutes pre-

cyclophosphamide, and post-cyclophosphamide hydration with 1/4-1/5 normal saline (30-50 ml/kg total volume); for patients with hypertension, volume overload or active nephrotic syndrome, fluid restriction (<1000 ml/day) was implemented with furosemide supplementation to maintain adequate urine output. 2) Prior to cyclophosphamide administration, maintain WBC $\geq 3.0\times 10^9$/L and lymphocytes $\geq 0.5\times 10^9$/L. If subthreshold, administer Leucogen tablets and recheck in 1-2 weeks. Resume cyclophosphamide only when criteria are met, with the delay not exceeding 14 days from the originally scheduled administration time.]

(3) Basic treatment

Glucocorticoids: Intravenous methylprednisolone pulse therapy should be administered at 15–30 mg/kg/day (maximum 500 mg/day) for three consecutive days weekly over two weeks following the diagnosis of proliferative LN. Following the pulse therapy interval and completion of the pulse therapy regimen, treatment should transition to oral prednisone (2 mg/kg/day, not exceeding 60 mg/day), which was then rapidly tapered by 24 weeks (Table 1 showed detailed information on glucocorticoids dosing and tapering schedules).

Hydroxychloroquine: Oral administration. 4-6 mg/kg/day, divided into two doses (must undergo fundus screening prior to treatment).

Angiotensin-converting enzyme inhibitors: Oral administration. Enalapril is initiated at 0.1 mg/(kg·d) (maximum 0.75 mg/(kg·d)), administered once daily or in two divided doses; Benazepril is initiated at 0.1 mg/(kg·d) (maximum 0.3 mg/(kg·d)), given once daily or divided into two doses; Fosinopril is started at 0.3 mg/(kg·d) (maximum 1.0 mg/(kg·d)) with once-daily administration; other ACE inhibitors may be considered as alternatives. The treatment should be discontinued if hypotensive reactions or persistent dry cough occur.

Table 1 Standardized prednisone dosing regimen.

time	Weight ≥40kg	Weight 30~＜40kg	Weight 25~＜30kg	Weight 20~＜25kg
Week 1	60 mg#	60 mg#	50 mg#	40 mg#
Week 2	60 mg#	60 mg#	50 mg#	40 mg#
Week 3	50 mg	50 mg	40 mg	35 mg
Week 4	50 mg	50 mg	40 mg	35 mg
Week 5-6	40 mg	40 mg	35 mg	30 mg
Week 7-8	40 mg	40 mg	35 mg	30 mg
Week 9-10	35 mg	30 mg	30 mg	25 mg
Week 11-12	35 mg	30 mg	30 mg	25 mg
Week 13-14	30 mg	25 mg	20 mg	20 mg
Week 15-16	30 mg	25 mg	20 mg	20 mg
Week 17-18	25 mg	20 mg	15 mg	15 mg
Week 19-20	25 mg	20 mg	15 mg	15 mg
Week 21-22	20 mg	15 mg	10 mg	10 mg
Week 23-24	20 mg	15 mg	10 mg	10 mg

#Patients were administered high-dose methylprednisolone infusions (15–30 mg/kg per dose, with a maximum of 500 mg per day) three times per week for two consecutive weeks following the diagnosis of proliferative LN. Following the pulse therapy interval and completion of the pulse therapy regimen, treatment should transition to oral prednisone.

5.3 Concomitant medication

(1) Calcium supplements, vitamin D, and active vitamin D analogs are allowed for use during this research.

(2) Various anticoagulant drugs, liver protection drugs and antibiotic are allowed for use during this research.

(3) Immunosuppressants other than the test drug are prohibited for use.

(4) Statins, plasma exchange, biological agents and high-dose intravenous immunoglobulin (total dose >2 g/kg) are prohibited for treatment.

(5) Dihydropyridine calcium antagonists, β-receptor blocker, diuretic and/or α-receptor blocker can be used for control, with a target blood pressure of: <120/80 mmHg.

(6) If blood glucose rises during treatment, the blood glucose shall be actively

controlled with hypoglycemic drugs.

(7) Various Chinese herbal medicines are prohibited for the treatment of kidney diseases during this research.

(8) For the other drugs or treatments that must be continued for the complicated diseases, the drug name (or other treatment name), dosage, times of use and time must be recorded on the Clinical Study Report for analysis and reporting during summary.

6. Test process and steps

The selected patients are randomly divided into two groups. Patients in MMF Group receive mycophenolate mofetil tablets and steroids for 24 weeks; and patients in IVC Group receive cyclophosphamide and steroids for 24 weeks. Both groups follow the "clinical trial flow chart" in operation.

7. Evaluation of efficacy and safety

7.1 Efficacy outcomes

Primary observation outcomes: The primary endpoint was the proportion of subjects achieving renal response at week 24, defined as the composite of complete renal response (CRR), primary efficacy renal response (PERR), and partial renal response (PRR).

Secondary observation outcomes: The change in the SLE disease activity index-2000 (SLEDAI-2K) score from baseline.

7.2 Efficacy criteria

- Complete renal response (PRR) [16]is defined as proteinuria < 0.5g/1.73 m^2 per day or <300mg/m^2 per day based on a 24-hour urine specimen, along with stabilization or improvement in kidney function (±10%-15% of baseline).
- Primary efficacy renal response (PERR) [16] was defined as a protein-creatinine ratio

(PCR) < 700 mg/g and an estimated glomerular filtration rate (eGFR) no worse than 20% below the pre-flare value or ≥ 60ml/min per 1.73 m^2.

- Partial renal response (PRR) [16] was defined as a reduction in proteinuria by at least 50%, PCR < 3000 mg/g and stabilization or improvement in kidney function (±10%-15% of baseline).
- No renal response (NRR) [16] was defined as failure to achieve either a partial or complete renal response.

7.3 Safety evaluation

- Changes of vital signs: Blood pressure, respiration and heart rate.
- Various indexes in laboratory examination: Blood and urine routine examination, liver function, renal function examination.
- Documentation of extrarenal lupus manifestations.
- Clinically reported adverse events and adverse reactions.

7.4 Exploratory Outcomes:

Efficacy outcomes at 12 weeks, renal response of different subgroups (participants with nephrotic-range proteinuria, class IV or IV+V lupus nephritis, class III or III+V lupus nephritis, class III+V or IV+V lupus nephritis, class III or IV lupus nephritis).

8. Reporting adverse events

8.1 Definition of adverse events

Adverse events (AEs) and serious adverse events (SAEs) in strict accordance with ICH E6 (R3) guidelines.

- **Adverse Event (AE)**: Any unfavourable medical occurrence in a trial participant administered the investigational product. The adverse event does not necessarily have a causal relationship with the treatment.
- **Serious Adverse Event (SAE)**: Any unfavourable medical occurrence that is considered serious at any dose if it: (1) Results in death, (2) Is life-threatening, (3)

Requires inpatient hospitalization or prolongation of existing hospitalization, (4) Results in persistent or significant disability/incapacity, or (5) An important medical event that may not be immediately life-threatening or result in death or hospitalisation, that may jeopardise the participant or that may require intervention to prevent serious outcomes.

8.1.1 Concerned adverse reactions of test drug MMF

- **Infection:** Patients receiving MMF are at an increased risk of developing bacterial, fungal, protozoal and new or reactivated viral infections, including opportunistic infections. Serious life-threatening infections, such as meningitis and infective endocarditis, are occasionally reported, and some evidences show that certain types of infections, such as conjugated and atypical waste bacilli infections, lay have a high incidence.
- **Gastrointestinal adverse reactions:** Gastrointestinal adverse reactions include nausea, vomiting, dyspepsia, abdominal pain, constipation, upper and lower gastrointestinal discomfort, gastrointestinal inflammation, gastroduodenal ulcer, gastrointestinal bleeding, ulceration and perforation, etc. Colitis (sometimes caused by cytomegalovirus), pancreatitis, and individual cases of intestinal villus atrophy. Digestive:
- **Blood system and immune system:** MMF can cause neutropenia, pure red cell aplasia (PRCA), acute inflammatory syndrome, hypersensitivity, and hypogammaglobulinemia have been reported.
- **Malignant tumors:** Patients receiving MMF are at increased risk of developing lymphoproliferative disease, lymphomas and other malignancies tumors, especially skin tumors.
- **Respiratory:** MMF can cause bronchiectasis, interstitial lung disease, fatal pulmonary fibrosis, have been reported rarely and should be considered in the differential diagnosis of pulmonary symptoms ranging from dyspnea to respiratory failure in post-transplant patients receiving mycophenolate mofetil.

- **Vascular:** MMF can cause lymphocele, venous thrombosis.

8.1.2 Concerned adverse reactions of test drug cyclophosphamide

- **Myelosuppression, Immunosuppression, Bone Marrow Failure and Infections:** Cyclophosphamide can cause myelosuppression (leukopenia, neutropenia, thrombocytopenia and anemia), bone marrow failure, and severe immunosuppression which may lead to serious and sometimes fatal infections, including sepsis and septic shock. Latent infections can be reactivated.
- **Urinary Tract and Renal Toxicity:** Cyclophosphamide can cause hemorrhagic cystitis, pyelitis, ureteritis, and hematuria, renal tubular disorder, nephrogenic diabetes insipidus,
- **Cardiotoxicity:** Cyclophosphamide can cause cardiomyopathy, pericarditis, carditis, myopericarditis, pericardial effusion including cardiac tamponade, and congestive heart failure, supraventricular arrhythmias (including atrial fibrillation and flutter) and ventricular arrhythmias (including severe QT prolongation associated with ventricular tachyarrhythmia).
- **Pulmonary Toxicity:** Cyclophosphamide can cause pulmonary fibrosis, pulmonary veno-occlusive disease, interstitial lung disease, obliterative bronchiolitis, organizing pneumonia, alveolitis allergic, pulmonary hemorrhage, pulmonary edema, bronchospasm, and other forms of pulmonary toxicity leading to respiratory failure have been reported during and following treatment with cyclophosphamide.
- **Secondary Malignancies:** Cyclophosphamide can cause secondary malignancies (urinary tract cancer, myelodysplasia, acute leukemias, lymphomas, thyroid cancer, and sarcomas) have been reported in patients treated with cyclophosphamide-containing regimens.
- **Ear and Labyrinth:** Cyclophosphamide can cause deafness, hearing impaired, tinnitus.
- **Eye:** Cyclophosphamide can cause visual impairment, conjunctivitis, lacrimation.
- **Gastrointestinal:** Cyclophosphamide can cause gastrointestinal hemorrhage, acute

pancreatitis, colitis, enteritis, cecitis, stomatitis, constipation, parotid gland inflammation, nausea, vomiting, diarrhea.

- **Hepatic:** Cyclophosphamide can cause veno-occlusive liver disease, cholestatic hepatitis, cytolytic hepatitis, hepatitis, cholestasis; blood bilirubin increased, hepatic function abnormal, hepatic enzymes increased.
- **Musculoskeletal and Connective Tissue:** Cyclophosphamide can cause rhabdomyolysis, scleroderma, muscle spasms, myalgia, arthralgia.
- **Nervous System:** Cyclophosphamide can cause encephalopathy, convulsion, dizziness, neurotoxicity has been reported and manifested as reversible posterior leukoencephalopathy syndrome, myelopathy, peripheral neuropathy, polyneuropathy, neuralgia, dysesthesia, hypoesthesia, paresthesia, tremor, dysgeusia, hypogeusia, parosmia.
- **Reproductive System:** Cyclophosphamide can cause infertility, ovarian failure, ovarian disorder, amenorrhea, oligomenorrhea, testicular atrophy, azoospermia, oligospermia. Embryo-Fetal Toxicity.
- **Skin and Subcutaneous Tissue:** Cyclophosphamide can cause toxic epidermal necrolysis, Stevens-Johnson syndrome, erythema multiforme, palmar-plantar erythrodysesthesia syndrome, radiation recall dermatitis, toxic skin eruption, urticaria, dermatitis, blister, pruritus, erythema, nail disorder, facial swelling, hyperhidrosis, alopecia.
- **Vascular:** Cyclophosphamide can cause pulmonary embolism, venous thrombosis, vasculitis, peripheral ischemia, hypertension, hypotension, flushing, hot flush.
- **Endocrine:** Hyponatremia, water intoxication, and a syndrome resembling syndrome of inappropriate secretion of antidiuretic hormone (SIADH).

8.2 Causality assessment

The WHO-UMC causality assessment system was employed for standardized case evaluation, with detailed causality categories presented in Table 2.

Table 2. WHO-UMC Causality Categories

Causality term	Assessment criteria
Certain	• Event or laboratory test abnormality, with plausible time relationship to drug intake<br>• Cannot be explained by disease or other drugs<br>• Response to withdrawal plausible (pharmacologically, pathologically)<br>• Event definitive pharmacologically or phenomenologically (i.e. an objective and specific medical disorder or a recognised pharmacological phenomenon)<br>• Rechallenge satisfactory, if necessary
Probable / Likely	• Event or laboratory test abnormality, with reasonable time relationship to drug intake<br>• Unlikely to be attributed to disease or other drugs<br>• Response to withdrawal clinically reasonable<br>• Rechallenge not required
Possible	• Event or laboratory test abnormality, with reasonable time relationship to drug intake<br>• Could also be explained by disease or other drugs<br>• Information on drug withdrawal may be lacking or unclear
Unlikely	• Event or laboratory test abnormality, with a time to drug intake that makes a relationship improbable (but not impossible)<br>• Disease or other drugs provide plausible explanations
Conditional / Unclassified	• Event or laboratory test abnormality<br>• More data for proper assessment needed, or<br>• Additional data under examination
Unassessable / Unclassifiable	• Report suggesting an adverse reaction<br>• Cannot be judged because information is insufficient or contradictory<br>• Data cannot be supplemented or verified

8.3 Records of adverse events

8.3.1 Components of adverse event data collection

- Endpoint event
- Adverse events related to the test drugs (according to the judgment of the investigators)
- Serious adverse event

8.3.2 Cycle of adverse event collection

- The collection of AEs and drug adverse events (DAEs) of concern will be carried

out throughout this research, from randomization to site close-out visit.

- SAE records will be carried out throughout this research, from the time of signing the Informed Consent Form to site close-out visit.

8.3.3 Follow-up of unresolved adverse events

If any AE, DAE and SAE of concern remain unresolved at the patient's last study visit, the investigators shall follow up these events in accordance with the medical instructions as required for medical needs in this research, but it is not necessary to record further in the electronic case report form (eCRF). If deemed necessary, the sponsor reserves the right to obtain the additional information on unresolved AE/SAE of concern.

8.3.4 Variables

For each non-serious AE and DAE of concern, the following variables will be collected:

- AE (verbatim record);
- AE start and end dates;
- Maximum intensity;
- Whether the AE is serious;
- The investigators' assessment of the causal relationship with the test drug (yes or no);
- Measures and outcomes taken for the test drug

In addition, the following variables will be collected for SAE:

- Date when AE meets SAE criteria;
- Date when the investigators find SAE;
- Reasons for classifying AE as SAE;
- Date of admission;
- Date of discharge;
- Possible causes of death;
- Date of death;

- Autopsy performed (if any);
- Autopsy results;
- AE description;
- Assessment of causality with this research procedure;
- Assessment of causality with the other drugs.

It is important to distinguish serious AE from SAE. Severity is the index of intensity evaluation, while seriousness is defined according to the criteria in Section 8.1. AE with severe intensity is not necessarily serious. For example, nausea for several hours may be considered as severe nausea, but not SAE. On the other hand, a stroke that results in only a limited degree of disability may be considered as a mild stroke, but should be considered as SAE.

8.4.5 Adverse events based on signs and symptoms

All concerned AEs (see sections 8.1.1 and 8.1.2), including those spontaneously reported by the patients, those reported in response to the open-ended question by the investigators, "Have you had any health problems since the last visit/inquiry?", or those found through observation will be all collected and recorded in eCRF. In the collection of AE information, the list of signs and symptoms shall be preferred as the record of diagnosis (if possible). However, if the diagnosis is known and there are other signs or symptoms not usually present in the diagnosis, the diagnosis and each other sign or symptom shall be recorded separately.

8.4.6 Adverse events based on examination and inspection

During this research, the laboratory safety assessment will not be carried out routinely (except at baseline), but can be carried out at the discretion of the investigators. When an AE, DAE, SAE or potential endpoint of concern is involved, different assessments may be required, including local laboratory assessment, and appropriate collection is needed before entry into eCRF.

The examination results of vital signs mandated by the Protocol shall be summarized

in the clinical study report. Therefore, when the laboratory test values and protocol-mandated vital signs evaluation by the investigators show that the results are worse than the baseline level and meet the AE criteria or when they act as are the reasons for discontinuing the test drug treatment, such situations will be reported as AE.

If the deterioration of laboratory test values/vital signs is related to clinical signs and symptoms, the signs and symptoms will be reported as AE and relevant laboratory results/vital signs will be used as supplementary information. Investigators shall use clinical terms rather than laboratory terms (e.g. renal failure rather than elevated creatinine) as much as possible in the report. If no clinical signs or symptoms are present, the clinically relevant deterioration of non-mandatory parameters shall be reported as AE and SAE.

8.4 Report of SAEs

All SAEs must be reported, regardless of whether they are considered to have causalities with the test drugs or study procedure. All SAEs will be recorded in eCRF.

In case of any SAE during this research, the investigators or other staff of the center shall notify the relevant Sponsor representative immediately or no later than 24 hours after learning of the event.

The designated Sponsor representative shall work with the investigators to ensure that all necessary information is provided to the Sponsor's patient safety data entry center within 1 working day after the first receipt of fatal and life-threatening events and 5 working days after the first receipt of all other SAEs.

For fatal and life-threatening AEs, if important or relevant information is missing, immediate active follow-up is required. The investigators or other staff of the center shall notify the Sponsor representative of any follow-up information about the previously reported SAE immediately or no later than 24 hours after receiving the information.

9. Quality control and assurance of the trial

9.1 Quality control measures in laboratory

Each center can keep the test results for a long time;

Each center appoints a specialist from the Pediatric Rheumatology, Immunology, and Nephrology Department to be responsible for the laboratory inspection related matters for this project.

9.2 Quality control and assurance in the trial process

(1) All hospitals participating in the clinical trial shall adopt standard operating procedures to ensure the implementation of quality control and quality assurance systems for the clinical trial.

(2) In order to ensure the quality of the multicenter trial, the principals of the participating hospitals shall discuss and prepare the clinical study plan together before the formal trial starts. The relevant medical personnel participating in the trial are trained at the same time.

(3) The hospitals participating in the trial shall, in accordance with the uniform clinical research protocol, start and end the trial at the same time as possible.

(4) All observed results and abnormal findings in the clinical trial shall be carefully verified and recorded in time to ensure the reliability of the data. The recording and transfer of clinical data must be in the charge of experienced physicians and supervised or checked by special personnel to ensure the scientificity and accuracy of the data. Various conclusions of the clinical trial must be derived from the original data.

(5) In the summarization and analysis of the clinical trial results, standardized statistical analysis methods must be adopted, and personnel familiar with biostatistics should be invited for participation.

(6) In order to better supervise and inspect the trial, the research and development unit and the leading unit of this clinical trial shall carry out irregular inspection on each

clinical trial hospital. It aims to determine whether the implementation of the trial, data recording and analysis are consistent with the requirements specified in the Protocol, drug clinical trial management specifications and regulations.

9.3 Measures to improve subject compliance

To ensure that the enrolled patients can positively complete the trial, importance must be attached to the measures of subject compliance.

(1) For each enrolled patient, it is necessary to clarify the possible benefits and risks of the test drugs to them.
(2) The Informed Consent Form must be signed by each enrolled patient or his/her legal representative and guardian.
(3) Each enrolled patient must be required to use the test drug or control drug in a sufficient amount and an effective way each time.
(4) If it is found that any subject refuses to use drugs (including the control drug) in study, the ideological work shall be actively done for the subject and the guardian.

9.4 Quality control and quality assurance systems

The investigator shall perform their respective responsibilities, strictly follow the clinical trial protocol and adopt standard operating procedures to ensure the implementation of quality control and quality assurance systems for the clinical trial.

The investigators shall follow the principles of GCP in the whole process of this research, and shall record all the content in CRF in a truthful, detailed and careful manner. All the observations and findings in the clinical trial shall be verified to ensure the reliability of the data.

10. Data management

The investigators shall ensure that the data are recorded in the electronic case report form (eCRF) in a timely manner in accordance with the provisions of the Protocol and the instructions provided.

The research staff will receive training and be responsible for inputting the data specified in the Protocol into the Excel Spreadsheet according to the eCRF instructions.

11. Statistical analysis

11.1 Analysis data set

Intention-to-treat (ITT) population: The ITT population included all randomised subjects, whether or not they followed the planned treatment process.

Per-protocol (PP) population: The PP population included subjects who strictly adhered to the experimental protocol. For instance, subjects lost to follow-up are excluded from this set.

11.2 Statistical method

11.2.1 Descriptive statistics

Continuous variables are presented as mean ± standard deviation (SD) if normally distributed, or as median (P25, P75) if not normally distributed. Categorical variables are described using number (percentage).

11.2.2 Between-group difference values

Between-group comparisons were reported as mean differences (MD) or rate differences (RD) with corresponding 95% confidence intervals (CIs), calculated using t-tests and the Newcombe-Wilson method (Figure 1), respectively.

$$\frac{2np + z^2 \pm z\sqrt{z^2 + 4npq}}{2(n + z^2)}$$

$$L = p_1 - p_2 - \sqrt{(p_1 - l_1)^2 + (u_2 - p_2)^2}$$

$$U = p_1 - p_2 - \sqrt{(p_2 - l_2)^2 + (u_1 - p_1)^2}$$

Figure 1. Newcombe-Wilson method

Where, q=1-p and z=1.96, at the same time, the upper and lower limits for rates of the two groups (p_1 and p_2), l_1, u_1, l_2, u_2, can be calculated by the first formula.

11.2.3 Type I error level

For the non - inferiority test, the significance level (α) is set at 0.025 (one - sided). For the difference test, a two - sided approach is adopted with a significance level of 0.05.

11.2.4 Non-inferiority margin

The fixed-margin method was employed to establish the non-inferiority margin. As no meta-analyses currently compare cyclophosphamide + glucocorticoid versus placebo + glucocorticoid for lupus nephritis, we referenced a randomized trial[17] reporting renal remission rates of 85% (17/20) with combination therapy (cyclophosphamide + glucocorticoid) versus 29% (7/24) with methylprednisolone alone (rate difference 56%, 95% CI 27-73%). Preserving 50-80% of cyclophosphamide's treatment effect and applying the formula (1 - preservation fraction) × 27%, yielded a margin range of 5.4-13.5%. Considering recruitment challenges following China's 2022 COVID-19 policy adjustments, we selected 12% as the non-inferiority margin.

11.2.5 Handling of Missing Data

Missing data were handled via last observation carried forward for intention-to-treat analysis.

11.2.6 Interim Analysis

When all enrolled patients completed the 3-month follow-up, data collected during this period were used for the interim analysis.

11.2.7 Primary efficacy analysis

Between-group non-inferiority in renal response was assessed using Farrington-Manning tests.

11.2.8 Secondary efficacy analysis

Differences between groups in changes of SLEDAI-2K scores from baseline were analyzed with the Student's t-test.

11.2.9 Safety analysis

Between-group differences in drug adverse events were assessed with chi-squared tests.

11.3 Data analysis software

Statistical analysis was performed using Statistical Analysis System software (version 9.4; SAS Institute, Cary, NC) and SPSS Statistics (version 26.0; IBM Corp., Armonk, NY, United States).

12. Ethical principles

12.1 Ethical review

The clinical trial protocol shall be submitted to the Ethics Committee for approval before implementation.

12.2 Benefits and risks

This test drug in this research is already on the market, and no serious adverse reactions have been reported in clinical application. In case of possible side effects, the investigators shall take the corresponding medical countermeasures according to the specific conditions of the patients and shall have the right to suspend the clinical trial based on their own judgment.

12.3 Informed consent process

The investigators must explain to the subjects about the details of the clinical trial, including its purpose, procedure, possible benefits and risks, the rights and obligations of the subjects, etc., so that the subjects can fully understand it and have plenty of time for consideration. The clinical trial can be started after the subjects get satisfactory answers to the questions raised, and sign the Informed Consent Form upon agreement. Whenever a patient signs the Informed Consent Form, the physician shall leave his/her contact number to the patient so that the patient can find him/her at any time if any condition change occurs. Informed Consent Form shall be submitted to the Ethics

Committee for review.

12.4 Medical treatment and protection of subjects

The investigators are responsible for the medical treatment of the subjects, making medical decisions related to the clinical trial, and ensuring that the subjects could receive appropriate treatment in case of adverse events during the trial.

12.5 Protection of subject privacy

Only the investigators involved in the clinical trial may have access to the personal medical records of the subjects. During data processing, the method of "data anonymity" is adopted, and the information that can identify the individual identity of the subjects will be omitted.

13. Ethical requirements

The clinical trial must be conducted in accordance with the Declaration of Helsinki and the relevant clinical trial research norms and regulations in China. The clinical trial can be implemented after the Protocol is approved by the Ethics Committee of the unit in charge of the clinical research.

Before each patient is enrolled in this research, the research physician has the responsibility to completely and comprehensively introduce the purpose, procedure and possible risks of this research to the patient or his/her designated representative in a written form, and let the patients know that they have the right to withdraw from this research at any time. Before enrollment, each patient or his family members must be given a written Informed Consent Form. The research physician has the responsibility to obtain the Informed Consent before each patient is included in this research. The Informed Consent Form shall be kept as a clinical trial document for future reference.

14. Data preservation

The trial protocol and its amendment (signed), Informed Consent Form (format),

approval from the Ethics Committee, membership list of the Ethics Committee, original outpatient or inpatient medical record, case report form (filled in, signed and dated), original of adverse event report and Excel Spreadsheet.

15. Follow-up and medical measures after the trial

If the subjects have adverse events/reactions during the clinical trial, they must be followed up within 1 month after the treatment to ensure their safety. If the subjects still need therapy for their uncured conditions after the end of the trial, the current standard therapeutic drugs can be used for treatment.

16. References

[1] Durcan L, O'Dwyer T, Petri M. Management strategies and future directions for systemic lupus erythematosus in adults. *Lancet*. 2019;393(10188):2332-2343. doi:10.1016/S0140-6736(19)30237-5

[2] Stojan G, Petri M. Epidemiology of systemic lupus erythematosus: an update. *Curr Opin Rheumatol*. 2018;30(2):144-150. doi:10.1097/BOR.000000000000048 0

[3] Tunnicliffe DJ, Palmer SC, Henderson L, et al. Immunosuppressive treatment for proliferative lupus nephritis. *Cochrane Database Syst Rev*. 2018;6(6):CD002922. doi:10.1002/14651858.CD002922.pub4

[4] Brunner HI, Gladman DD, Ibañez D, et al. Difference in disease features between childhood-onset and *adult*-onset systemic lupus erythematosus. *Arthritis Rheum*. 2008;58(2):556-562. doi:10.1002/art.23204

[5] Ardoin SP, Daly RP, Merzoug L, et al. Research priorities in childhood-onset lupus: results of a multidisciplinary prioritization exercise. *Pediatr Rheumatol Online J*. 2019;17(1):32. doi:10.1186/s12969-019-0327-4

[6] Weening JJ, D'Agati VD, Schwartz MM, et al. The classification of glomerulonephritis in systemic lupus erythematosus revisited. *J Am Soc Nephrol*. 2004;15(2):241-250. doi:10.1097/01.asn.0000108969.21691.5d

[7] Nephrology Group of the Pediatrics Branch of the Chinese Medical. Association Evidence-based Guidelines for the diagnosis and treatment of lupus nephritis (2016) [J]. *Chinese Journal of Pediatrics*, 2018, 56(2): 88-94. doi: 10.3760/cma. j.issn.0578-1310.2018.02.003.

[8] The Compilation Group of Guidelines for the Diagnosis and Treatment of Lupus Nephritis in China. Chinese Guidelines for the Diagnosis and Treatment of Lupus Nephritis [J]. Chinese Medical Journal. 2019,99(44):3441-3455. doi:10.3760/cma. j.issn.0376-2491.2019.44.001.

[9] Ginzler, Ellen M et al. "Mycophenolate mofetil or intravenous cyclophosphamide for lupus nephritis." *The New England journal of medicine* vol. 353,21 (2005): 2219-28. doi:10.1056/NEJMoa043731

[10] Appel, Gerald B et al. "Mycophenolate mofetil versus cyclophosphamide for induction treatment of lupus nephritis." *Journal of the American Society of Nephrology: JASN* vol. 20,5 (2009): 1103-12. doi:10.1681/ASN.2008101028

[11] Zhang H, Zhou M, Han X, et al. Mycophenolate mofetil in the treatment of Chinese patients with lupus nephritis: A PRISMA-compliant meta-analysis. *Medicine (Baltimore)*. 2020;99(33):e21121. doi:10.1097/MD.000000000002112

[12] Groot N, de Graeff N, Marks SD, et al. European evidence-based recommendations for the diagnosis and treatment of childhood-onset lupus nephritis: the SHARE initiative. *Ann Rheum Dis*. 2017;76(12):1965-1973. doi:10.1136/annrheumdis-2017-211898

[13] Lau KK, Ault BH, Jones DP, et al. Induction therapy for pediatric focal proliferative lupus nephritis: cyclophosphamide versus mycophenolate mofetil. *J Pediatr Health Care*. 2008;22(5):282-288. doi:10.1016/j.pedhc.2007.07.006

[14] Aringer M, Costenbader K, Daikh D, et al. 2019 European League Against Rheumatism/American College of Rheumatology Classification Criteria for Systemic Lupus Erythematosus. *Arthritis Rheumatol*. 2019;71(9):1400-1412. doi:10.1002/art.40930

[15] Petri M, Orbai AM, Alarcón GS, et al. Derivation and validation of the Systemic Lupus International Collaborating Clinics classification criteria for systemic lupus

erythematosus. *Arthritis Rheum*. 2012;64(8):2677-2686. doi:10.1002/art. 34473

[16] Kidney Disease: Improving Global Outcomes (KDIGO) Lupus Nephritis Work Group. KDIGO 2024 Clinical Practice Guideline for the management of LUPUS NEPHRITIS. *Kidney Int*. 2024;105(1S):S1-S69. doi:10.1016/j.kint.2023.09.002

[17] Gourley MF, Austin HA 3rd, Scott D, et al. Methylprednisolone and cyclophosphamide, alone or in combination, in patients with lupus nephritis. A randomized, controlled trial. *Ann Intern Med*. 1996;125(7):549-557. doi:10.7326/0003-4819-125-7-199610010-00003

Appendix

Appendix 1 2019 American College of Rheumatology classification criteria for SLE

Entry criterion
Antinuclear antibodies (ANA) at a titer of ≥1:80 on HEp-2 cells or an equivalent positive test (ever)

↓

If absent, do not classify as SLE
If present, apply additive criteria

↓

Additive criteria
Do not count a criterion if there is a more likely explanation than SLE.
Occurrence of a criterion on at least one occasion is sufficient.
SLE classification requires at least one clinical criterion and ≥10 points.
Criteria need not occur simultaneously.
Within each domain, only the highest weighted criterion is counted toward the total score§.

Clinical domains and criteria	Weight	Immunology domains and criteria	Weight
Constitutional		***Antiphospholipid antibodies***	
Fever	2	Anti-cardiolipin antibodies OR	
Hematologic		Anti-β2GP1 antibodies OR	
Leukopenia	3	Lupus anticoagulant	2
Thrombocytopenia	4	***Complement proteins***	
Autoimmune hemolysis	4	Low C3 OR low C4	3
Neuropsychiatric		Low C3 AND low C4	4
Delirium	2	***SLE-specific antibodies***	
Psychosis	3	Anti-dsDNA antibody* OR	
Seizure	5	Anti-Smith antibody	6
Mucocutaneous			
Non-scarring alopecia	2		
Oral ulcers	2		
Subacute cutaneous OR discoid lupus	4		
Acute cutaneous lupus	6		
Serosal			
Pleural or pericardial effusion	5		
Acute pericarditis	6		
Musculoskeletal			
Joint involvement	6		
Renal			
Proteinuria >0.5g/24h	4		
Renal biopsy Class II or V lupus nephritis	8		
Renal biopsy Class III or IV lupus nephritis	10		

Total score:

↓

Classify as Systemic Lupus Erythematosus with a score of 10 or more if entry criterion fulfilled.

Appendix 2 2012 Systemic Lupus International Collaborating Clinics criteria

Classify a patient as having SLE if they meet either of these conditions: (1) The patient satisfies four of the criteria listed in following table (including at least one clinical criterion and one immunologic criterion), or (2) The patient has biopsy-proven nephritis compatible with SLE and with ANA or anti-dsDNA antibodies.

**Clinical Criteria**
1. Acute cutaneous lupus
including lupus malar rash (do not count if malar discoid)
bullous lupus
toxic epidermal necrolysis variant of SLE
maculopapular lupus rash
photosensitive lupus rash
in the absence of dermatomyositis
or subacute cutaneous lupus
(nonindurated psoriaform and/or annular polycyclic lesions that resolve without scarring, although occasionally with postinflammatory dyspigmentation or telangiectasias)
2. Chronic cutaneous lupus
including classical discoid rash
localized (above the neck)
generalized (above and below the neck)
hypertrophic (verrucous) lupus
lupus panniculitis (profundus)
mucosal lupus
lupus erythematosus tumidus
chillblains lupus
discoid lupus/lichen planus overlap
3. Oral ulcers: palate
buccal
tongue
or nasal ulcers
in the absence of other causes, such as vasculitis, Behcets, infection (herpes), inflammatory bowel disease, reactive arthritis, and acidic foods
4. Nonscarring alopecia (diffuse thinning or hair fragility with visible broken hairs)
in the absence of other causes such as alopecia areata, drugs, iron deficiency and androgenic alopecia
5. Synovitis involving two or more joints, characterized by swelling or effusion OR tenderness in 2 or more joints and thirty minutes or more of morning stiffness.
6. Serositis
typical pleurisy for more than 1 day
or pleural effusions
or pleural rub
typical pericardial pain (pain with recumbency improved by sitting forward) for more than 1 day
or pericardial effusion
or pericardial rub
or pericarditis by EKG
in the absence of other causes, such as infection, uremia, and Dressler's pericarditis
7. Renal
Urine protein/creatinine (or 24 hr urine protein) representing 500 mg of protein/24 hr

Clinical Criteria
or
Red blood cell casts
8. Neurologic
seizures
psychosis
mononeuritis multiplex
in the absence of other known causes such as primary vasculitis
myelitis
peripheral or cranial neuropathy
in the absence of other known causes such as primary vasculitis, infection, and diabetes mellitus
acute confusional state
in the absence of other causes, including toxic-metabolic, uremia, drugs
9. Hemolytic anemia
10. Leukopenia (< 4000/mm^3 at least once)
in the absence of other known causes such as Felty's, drugs, and portal hypertension
OR
Lymphopenia (< 1000/mm^3 at least once)
in the absence of other known causes such as corticosteroids, drugs and infection
11. Thrombocytopenia (<100,000/mm^3) at least once
in the absence of other known causes such as drugs, portal hypertension, and TTP

Immunological Criteria
1. ANA above laboratory reference range
2. Anti-dsDNA above laboratory reference range, except ELISA: twice above laboratory reference range
3. Anti-Sm
4. Antiphospholipid antibody: any of the following
lupus anticoagulant
false-positive RPR
medium or high titer anticardiolipin (IgA, IgG or IgM)
anti-β_2 glycoprotein I (IgA, IgG or IgM)
5. Low complement
low C3
low C4
low CH50
6. Direct Coombs test *in the absence of hemolytic anemia*

Criteria are cumulative and need not be present concurrently.

Appendix 3 classification of lupus nephritis according to the 2003 ISN/RPS;

Table 3. International Society of Nephrology/Renal Pathology Society (ISN/RPS) 2003 classification of lupus nephritis

**Class I**	**Minimal mesangial lupus nephritis**<br>Normal glomeruli by light microscopy, but mesangial immune deposits by immunofluorescence
**Class II**	**Mesangial proliferative lupus nephritis**<br>Purely mesangial hypercellularity of any degree or mesangial matrix expansion by light microscopy, with mesangial immune deposits<br>May be a few isolated subepithelial or subendothelial deposits visible by immunofluorescence or electron microscopy, but not by light microscopy
**Class III**	**Focal lupus nephritis**[a]<br>Active or inactive focal, segmental or global endo- or extracapillary glomerulonephritis involving <50% of all glomeruli, typically with focal subendothelial immune deposits, with or without mesangial alterations
Class III (A)	Active lesions: focal proliferative lupus nephritis
Class III (A/C)	Active and chronic lesions: focal proliferative and sclerosing lupus nephritis
Class III (C)	Chronic inactive lesions with glomerular scars: focal sclerosing lupus nephritis
**Class IV**	**Diffuse lupus nephritis**[b]<br>Active or inactive diffuse, segmental or global endo- or extracapillary glomerulonephritis involving ≥50% of all glomeruli, typically with diffuse subendothelial immune deposits, with or without mesangial alterations. This class is divided into diffuse segmental(IV-S) lupus nephritis when ≥50% of the involved glomeruli have segmental lesions, and diffuse global (IV-G) lupus nephritis when ≥50% of the involved glomeruli have global lesions. Segmental is defined as a glomerular lesion that involves less than half of the glomerular tuft. This class includes cases with diffuse wire loop deposits but with little or no glomerular proliferation
Class IV-S (A)	Active lesions: diffuse segmental proliferative lupus nephritis
Class IV-G (A)	Active lesions: diffuse global proliferative lupus nephritis
Class IV-S (A/C)	Active and chronic lesions: diffuse segmental proliferative and sclerosing lupus nephritis
	Active and chronic lesions: diffuse global proliferative and sclerosing lupus nephritis
Class IV-S (C)	Chronic inactive lesions with scars: diffuse segmental sclerosing lupus nephritis
Class IV-G (C)	Chronic inactive lesions with scars: diffuse global sclerosing lupus nephritis
**Class V**	**Membranous lupus nephritis**<br>Global or segmental subepithelial immune deposits or their morphologic sequelae by light microscopy and by immunofluorescence or electron microscopy, with or without mesangial alterations<br>Class V lupus nephritis may occur in combination with class III or IV in which case both will be diagnosed<br>Class V lupus nephritis show advanced sclerosis
**Class VI**	**Advanced sclerosis lupus nephritis**<br>≥90% of glomeruli globally sclerosed without residual activity

[a] Indicate the proportion of glomeruli with active and with sclerotic lesions.
[b] Indicate the proportion of glomeruli with fibrinoid necrosis and/or cellular crescents.
Indicate and grade (mild, moderate, severe) tubular atrophy, interstitial inflammation and fibrosis, severity of arteriosclerosis or other vascular lesions.

Appendix 4 SLEDAI-2K

Table 2. SLEDAI– 2K data collection form.

Study No.: ___________ Patient Name: ____________________ ______________ Visit Date: ___

(Enter weight in SLEDAI Score column if descriptor is present at the time of the visit or in the preceding 10 days.)

Weight	SLEDAI SCORE	Descriptor	Definition
8	________	Seizure	Recent onset, exclude metabolic, infectious or drug causes.
8	________	Psychosis	Altered ability to function in normal activity due to severe disturbance in the perception of reality. Include hallucinations, incoherence, marked loose associations, impoverished thought content, marked illogical thinking, bizarre, disorganized, or catatonic behavior. Exclude uremia and drug causes.
8	________	Organic brain syndrome	Altered mental function with impaired orientation, memory, or other intellectual function, with rapid onset and fluctuating clinical features, inability to sustain attention to environment, plus at least 2 of the following: perceptual disturbance, incoherent speech, insomnia or daytime drowsiness, or increased or decreased psychomotor activity. Exclude metabolic, infectious, or drug causes.
8	________	Visual disturbance	Retinal changes of SLE. Include cytoid bodies, retinal hemorrhages, serous exudate or hemorrhages in the choroid, or optic neuritis. Exclude hypertension, infection, or drug causes.
8	________	Cranial nerve disorder	New onset of sensory or motor neuropathy involving cranial nerves.
8	________	Lupus headache	Severe, persistent headache; may be migrainous, but must be nonresponsive to narcotic analgesia.
8	________	CVA	New onset of cerebrovascular accident(s). Exclude arteriosclerosis.
8	________	Vasculitis	Ulceration, gangrene, tender finger nodules, periungual infarction, splinter hemorrhages, or biopsy or angiogram proof of vasculitis.
4	________	Arthritis	$\geq$ 2 joints with pain and signs of inflammation (i.e., tenderness, swelling or effusion).
4	________	Myositis	Proximal muscle aching/weakness, associated with elevated creatine phosphokinase/aldolase or electromyogram changes or a biopsy showing myositis.
4	________	Urinary casts	Heme-granular or red blood cell casts.
4	________	Hematuria	>5 red blood cells/high power field. Exclude stone, infection or other cause.
4	________	Proteinuria	>0.5 gram/24 hours
4	________	Pyuria	>5 white blood cells/high power field. Exclude infection.
2	________	Rash	Inflammatory type rash.
2	________	Alopecia	Abnormal, patchy or diffuse loss of hair.
2	________	Mucosal ulcers	Oral or nasal ulcerations.
2	________	Pleurisy	Pleuritic chest pain with pleural rub or effusion, or pleural thickening.
2	________	Pericarditis	Pericardial pain with at least 1 of the following: rub, effusion, or electrocardiogram or echocardiogram confirmation.
2	________	Low complement	Decrease in CH50, C3, or C4 below the lower limit of normal for testing laboratory
2	________	Increased DNA binding	Increased DNA binding by Farr assay above normal range for testing laboratory.
1	________	Fever	>38° C. Exclude infectious cause.
1	________	Thrombocytopenia	<100,000 platelets / x10^9/L, exclude drug causes.
1	________	Leukopenia	< 3,000 white blood cells / x10^9/L, exclude drug causes.

TOTAL SLEDAI SCORE ________

Appendix 5 Estimation of glomerular filtration rate

1.The glomerular filtration rate (eGFR) is calculated and estimated with Schwartz formula:

eGFR (mL/min/1.73m^2) =0.413 (cm) ×height (cm)/Scr (mg/dL)

2. At the same time: Unit conversion for serum creatinine (SCR) value: 1 mg/dL = 88.4 μmol/L

REFERENCES

Schwartz GJ, Muñoz A, Schneider MF, et al. New equations to estimate GFR in children with CKD. *J Am Soc Nephrol*. 2009;20(3):629-637. doi:10.1681/ASN.2008030287

Appendix 6 Parameters and results sample size calculation

StatBox - A Online Statistical Computing System

2×2 contingency table chi-square test

Parameters and Computed Sample Size

π_t	π_c	Δ	n_c	N_{total}	Actual Power(1-β)
0.84	0.71	-0.12	44	88	0.8047

Elements of Hypothesis Testing

Hypothesis	α	β	Sides
$H_0 : \pi_t - \pi_c \leq -0.12$ | $H_1 : \pi_t - \pi_c > -0.12$	0.025	0.2	1-sided

ILLUSTRATE:
1. The required sample size for comparing proportions between two groups was calculated using Pearson's chi-square test with the normal approximation method.
2. A one-sided test.
3. The calculated results were consistent with those obtained from SAS 9.4 (using test=PCHI).

REFERENCES

1. Fleiss, J. L., Tytun, A., and Ury, H. K. (1980). "A Simple Approximation for Calculating Sample Sizes for Comparing Independent Proportions." Biometrics 36:343–346.2.
2. Diegert, C., and Diegert, K. V. (1981). "Note on Inversion of Casagrande-Pike-Smith Approximate Sample Size Formula for Fisher-Irwin Test on 2x2 Tables." Biometrics 37:595.

中南大学湘雅二医院临床研究伦理委员会
临床研究审查意见

（2022）伦审【临研】第（063）号

项目名称	儿童 SLE 精准诊疗体系的研究				
发起单位	中南大学湘雅二医院				
主要研究者	吴小川/主任医师		专业	儿科	
研究性质	☑ 回顾性 ☐ 诊断性 ☑ 干预性 ☑ 生物标本利用<br>☐ 预后观察性 ☐ 问卷调查 ☐ 其他				
经费来源	中央财政专项资金				
审查方式	■会议审查 ☐快速审查		审查地点	精卫楼 19 楼机构会议室	
委员人数	13 人	到会委员人数	13 人	回避委员	无
邀请专家	无	材料形式审查	■合格 ☐不合格		
审阅及审评文件	1. 临床试验方案（方案编号:2021YFC2702004、版本号:1.0、版本日期:2021.12.16)；<br>2. 知情同意申请书（版本号：1.0、版本日期：2021.12.16)；<br>3. 研究者简历；<br>4. 参加中心列表；<br>5. 病例报告表(版本号：1.0、版本日期：2021.12.16)；<br>6. 风险处置预案(版本号：1.0、版本日期：2021.12.16)；<br>7. 人类遗传资源管理承诺书；				
伦理委员会意见	■同意 ☐作必要修改后同意 ☐不同意 ☐终止或暂停已批准的试验<br>备注：				
跟踪审查频率	☐3 个月 ☐6 个月 ■12 个月 ☐不适用				
批件有效期	一年		批件失效日期	2023 年 3 月 29 日	
主任委员/授权副主任委员签名：[signature] 日期：2022.3.30<br>中南大学湘雅二医院临床研究伦理委员会（盖章）					

Page 1 of 2

中南大学湘雅二医院临床研究伦理委员会
伦理审评会签到表

会议时间： 2022 年 3 月 30 日　　　　会议地点：精卫楼 19 楼机构会议室

姓名	性别	职称	工作单位	签名
陈晋东（主任委员）	男	主任医师<br>教授	中南大学湘雅二医院	
唐罗生（副主任委员）	男	主任医师<br>教授	中南大学湘雅二医院	
罗爱静（委员）	女	主任医师<br>教授	中南大学湘雅二医院	
黄江生（委员）	男	主任医师<br>教授	中南大学湘雅二医院	
陶澄（委员）	男	主任医师<br>教授	中南大学湘雅二医院	
李霞（委员）	女	主任医师<br>教授	中南大学湘雅二医院	
吴尚洁（委员）	女	主任医师<br>教授	中南大学湘雅二医院	
张湘瑜（委员）	女	主任医师<br>教授	中南大学湘雅二医院	
张毕奎（委员）	男	主任药师<br>教授	中南大学湘雅二医院	
吴忠仕（委员）	男	主任医师<br>教授	中南大学湘雅二医院	
李卓（委员）	女	副主任医师<br>副教授	中南大学湘雅二医院	
任国峰（委员）	男	教授	中南大学公共卫生学院	
罗冰（委员）	女	律师	湖南弘湘律师事务所	

保密协议及利益冲突声明：作为中南大学湘雅二医院临床研究伦理委员会成员，我将对我所审阅的临床研究资料（详见审评文件目录）以及临床研究伦理委员会会议讨论的结果和相关内容保密，并保证所审查的项目与本临床研究伦理委员会成员无任何利益冲突。

中南大学湘雅二医院临床研究伦理委员会
审查意见

LYEC2024-K0022

**项目名称**	儿童 SLE 精准诊疗体系的研究		
**发起单位**	中南大学湘雅二医院	**申办方**	无
**经费来源**	纵向		
**院内编号**	LYG20220035(牵头)	**项目性质**	干预
**主要研究者**	吴小川	**专业**	儿科
**职称**	主任医师	**审查类型**	年度报告审查
**审查方式**	快速审查	**审查日期**	2024-04-01
**审查委员及意见**	李卓	☑同意 ☐不同意 ☐需会议审查	
	张毕奎	☑同意 ☐不同意 ☐需会议审查	
	备注：		
**签署**	03388479-2c49-4f4c-86fe-cb8f6a47f1bd<br>主任委员/授权副主任委员签名：[signature]<br>日期：2024-04-01<br>中南大学湘雅二医院临床研究伦理委员会（盖章）		
**跟踪审查频率**	☐3 个月 ☐6 个月 ☑12 个月 ☐其他		
**批件有效期**	☑批件失效日期 2025-03-31 ☐其他		
**附件**	审评文件目录		

**相关说明**	1、涉及人类遗传资源采集、保藏、利用、对外提供的研究项目，须获得中国人类遗传资源管理办公室的批准后开展研究；<br>2、研究过程中变更主要研究者，对研究方案、知情同意书、招募材料、给受试者的其他材料等的任何修改及变更，须及时向伦理委员会提交修正案审查申请；<br>3、请按照伦理委员会规定的年度/跟踪审查频率，在截止日期前一个月提交研究进展报告；当出现任何可能显著影响研究进行、或增加受试者风险的情况，请申请人及时向伦理委员会提交书面报告；<br>4、发生严重不良事件以及影响研究风险收益比的非预期不良事件，应及时报告伦理委员会及相关部门；<br>5、不依从或违背方案应及时通知伦理委员会并提交书面报告；<br>6、需暂停/提前终止/完成临床研究时请及时通知委员会并提交书面报告；<br>7、完成临床研究，请向委员会提交结题报告；<br>8、在临床试验过程中请严格遵循医学伦理等规范和原则，切实保障受试者权益；<br>9、研究结果不得直接作为注册申报使用；<br>10、联系方式：中南大学湘雅二医院临床研究伦理委员会<br>地址：湖南省长沙市人民中路 139 号<br>联系电话：0731-85292476

附件 1：

审评文件目录

03388479-2c49-4f4c-89fe-cb8f6a47f1bd

**项目名称**	儿童 SLE 精准诊疗体系的研究
**审查日期**	2024-04-01
1、中南大学湘雅二医院研究/年度进展报告（版本号：无，版本日期：2024-03-29）<br>2、延迟递交进展报告的情况说明（版本号：无，版本日期：2024-03-29）	

--------------------以上为所有审评文件，以下无正文--------------------
